# Two traditional Apulian varieties of *Cucumis melo* L. exhibit superior sensory and digestive profiles over common cucumber: a population-based and clinical study

**DOI:** 10.3389/fnut.2025.1662952

**Published:** 2025-10-21

**Authors:** Mohamad Khalil, Massimiliano Renna, Gianni Pietragalla, Valeria Perniola, Agostino Di Ciaula, Onofrio Davide Palmitessa, Adriano Didonna, Pietro Santamaria, Piero Portincasa

**Affiliations:** ^1^Clinica Medica “A. Murri”, Department of Precision and Regenerative Medicine and Ionian Area (DiMePrev-J), University of Bari Aldo Moro, Bari, Italy; ^2^Department of Soil, Plant and Food Sciences, University of Bari Aldo Moro, Bari, Italy

**Keywords:** *Cucumis*, gastrointestinal motility, sensory evaluation, food intake control, sustainable foods, Mediterranean diet

## Abstract

**Introduction:**

*Cucumis* vegetables, including the Apulian unripe melon varieties ‘Barattiere’ (BAR) and ‘Scopatizzo’ (SCO), represent important contributors to regional food agrobiodiversity.

**Methods:**

We conducted a population-based dietary survey (*N* = 493), sensory evaluations, and a clinical trial to compare consumption patterns, tolerability, and physiological responses of BAR and SCO with the commonly consumed cucumber (CUC).

**Results:**

Survey data revealed that CUC was the most consumed (98.4%), followed by BAR (92.7%) and SCO (79.5%). However, CUC intake was most frequently linked to digestive discomfort (16.3%) and maldigestion (11.5%), whereas BAR and SCO showed better gastrointestinal tolerance. Sensory testing identified BAR as the sweetest (*p* < 0.00001), SCO as the crunchiest (*p* = 0.002), and CUC as the most bitter (*p* < 0.00001). In the clinical trial (15 healthy adults, randomized crossover design), ingestion of 80 g portions demonstrated that BAR and SCO elicited stronger rewarding responses in food intake control (*p* < 0.02), while appetite and satiety parameters remained comparable across varieties. Functional ultrasonography and breath hydrogen tests showed mild and comparable gastrointestinal effects, without significant fermentation or bothersome symptoms.

**Discussion:**

Our findings demonstrate that BAR and SCO combine greater sensory appeal with improved digestive tolerance compared to CUC. These traditional varieties may therefore be promoted as culturally relevant, palatable, and physiologically favorable alternatives to common cucumbers. Further research is warranted to assess their broader metabolic and health effects.

## 1 Introduction

Promoting sustainable food choices is crucial not only for environmental preservation but also for improving public health and achieving global targets, as the United Nations Sustainable Development Goals (SDGs^1^), particularly SDG 2 (Zero Hunger) and SDG 3 (Good Health and Well-being) (1). As dietary patterns play a pivotal role in the prevention of non-communicable diseases, understanding how food sustainability, nutritional value, and cultural relevance can promote food choices is of key importance. Following the first representation of the traditional Mediterranean dietary model (2), there has been increasing interest in the contribution of agriculture and food production to climate change and, in general, to environmental damage, with a recently revised proposal of the diet pyramid (3). Valorizing traditional and locally adapted foods aligns with the broader objective of fostering resilient, health-promoting food systems that support both individual well-being (4) including metabolic and gastrointestinal health (5, 6).

Apulia is the Southern Italian region which boasts a remarkable richness in agrobiodiversity, and is an example of how local varieties (alias landraces or traditional varieties) of vegetables can successfully merge with modern horticultural practices (7). Notably, while global vegetable production largely relies on commercial hybrid varieties, local cultivars remain essential for preserving biodiversity, enriching regional cuisines, promoting sustainable farming practices, and combating malnutrition in meaningful, culturally relevant ways. Strengthening the connections between local food systems and the healthcare system could serve as a key strategy to address the dual challenges of both undernutrition and overnutrition (8).

Among the many vegetables cultivated in Puglia, particular relevance is attributed to species within the Cucurbitaceae family, notably those belonging to the genus *Cucumis*. Within this botanical *genus*, vegetables belonging to the species *Cucumis melo* L. are of great interest, especially some Apulian local varieties such as ‘Barattiere’ (BAR) and ‘Scopatizzo’ (SCO), whose fruits picked while still immature, meant to be eaten fresh in salads or plain, serving as a favorable alternative to cucumber (*Cucumis sativus* L.) because of their interesting quality traits and the absence of cucurbitacins. Indeed, Renna et al. (9) reported that these local varieties of *C. melo* exhibit lower levels of sugars and potassium compared to commercial cultivars of the same species, such as cantaloupe melon types. Therefore, the consumption of this local variety could be preferred to the cantaloupe melon when restrictions on high-K or sugar foods are recommended, such as for people with kidney disease or those with impaired insulin metabolism. At the same time, literature reports that the presence of cucurbitacins in the fruits of SCO and BAR has been detected only in rare cases (10). This aspect is particularly relevant if we consider that cucurbitacins are known for their bitterness and toxicity, and their presence in Cucurbitaceae species (such as *C. sativus*) is generally considered undesirable.

Moreover, it is important to highlight that these melon fruits are harvested before the seeds are fully developed and are marketed as “whole-edible” fruits, meaning that consumers enjoy not just the mesocarp but also the exocarp and placental portions. Nutritionally, the fact that the entire fruit can be consumed is significant, particularly because the placenta is rich in polyphenols and tocopherols, most notably *α*-tocopherol, which offer several health benefits (11).

In Apulia, people traditionally prefer to eat BAR and SCO instead of CUC, based on the popular claim that unripe melons are “easier to digest” and “better tolerated.” Although consumers often misuse the terms “digestibility” and “tolerance,” literature lacks comparative data on the digestibility of cucumbers versus unripe melons.

The gastrointestinal (GI) tract functions are the central interface between ingested foods and the human body, orchestrating complex processes of digestion, nutrient absorption, and signaling that are fundamental to maintaining metabolic health (12). Beyond its mechanical and enzymatic roles, the gut is a dynamic sensory and endocrine organ that communicates bidirectionally with the liver and the brain, influencing food perception, satiety, and overall energy homeostasis (13). Investigating gastrointestinal motility, digestion dynamics, and the sensory perception of foods provides critical insights into how specific food items interact with the body, thereby shaping eating behavior and nutritional outcomes (14–16).

Such an integrated approach is essential for evaluating the health potential of novel or traditional foods, optimizing dietary interventions, and addressing the growing burden of diet-related disorders, including malnutrition and metabolic disease.

This study aims to investigate the sensory characteristics, digestive responses, and potential for understanding the nutritional value of two traditional Apulian *Cucumis* varieties (i.e., BAR and SCO) in comparison with the commonly consumed cucumber (CUC). By integrating a population-based survey with clinical studies, we explore the consumption patterns, the postcibal gastrointestinal motility and subjective perceptions, to support the valorization of these heritage vegetables in both dietary and cultural contexts.

## 2 Materials and methods

**Figure 1:**
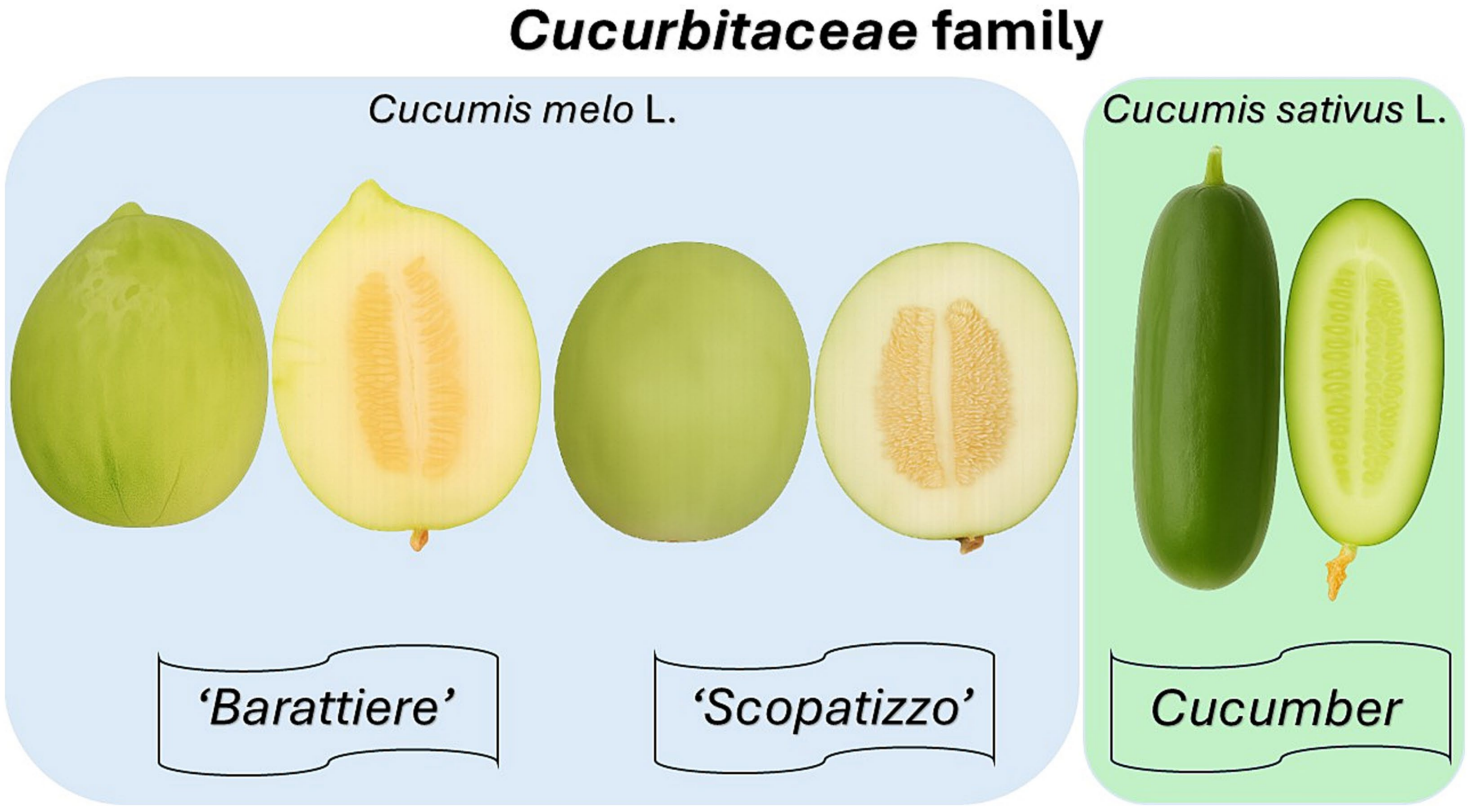
Visual comparison of the three *Cucumis* varieties analyzed in the study. From left to right: ‘Barattiere,’ ‘Scopatizzo,’ and cucumber are shown as whole and sliced fruits. ‘Barattiere’ and ‘Scopatizzo’ are traditional unripe melon landraces from the Apulian region, characterized by distinct morphological and sensory traits. ‘Barattiere’ typically has a spherical shape with light green flesh, while ‘Scopatizzo’ is more oval with firmer, crisp texture. The common cucumber, used as a reference, presents a darker green skin and a more watery internal structure. This image illustrates the external and internal variability among the varieties, which supports their differentiation in terms of sensory perception, nutritional value, and digestive responses.

**Figure 2:**
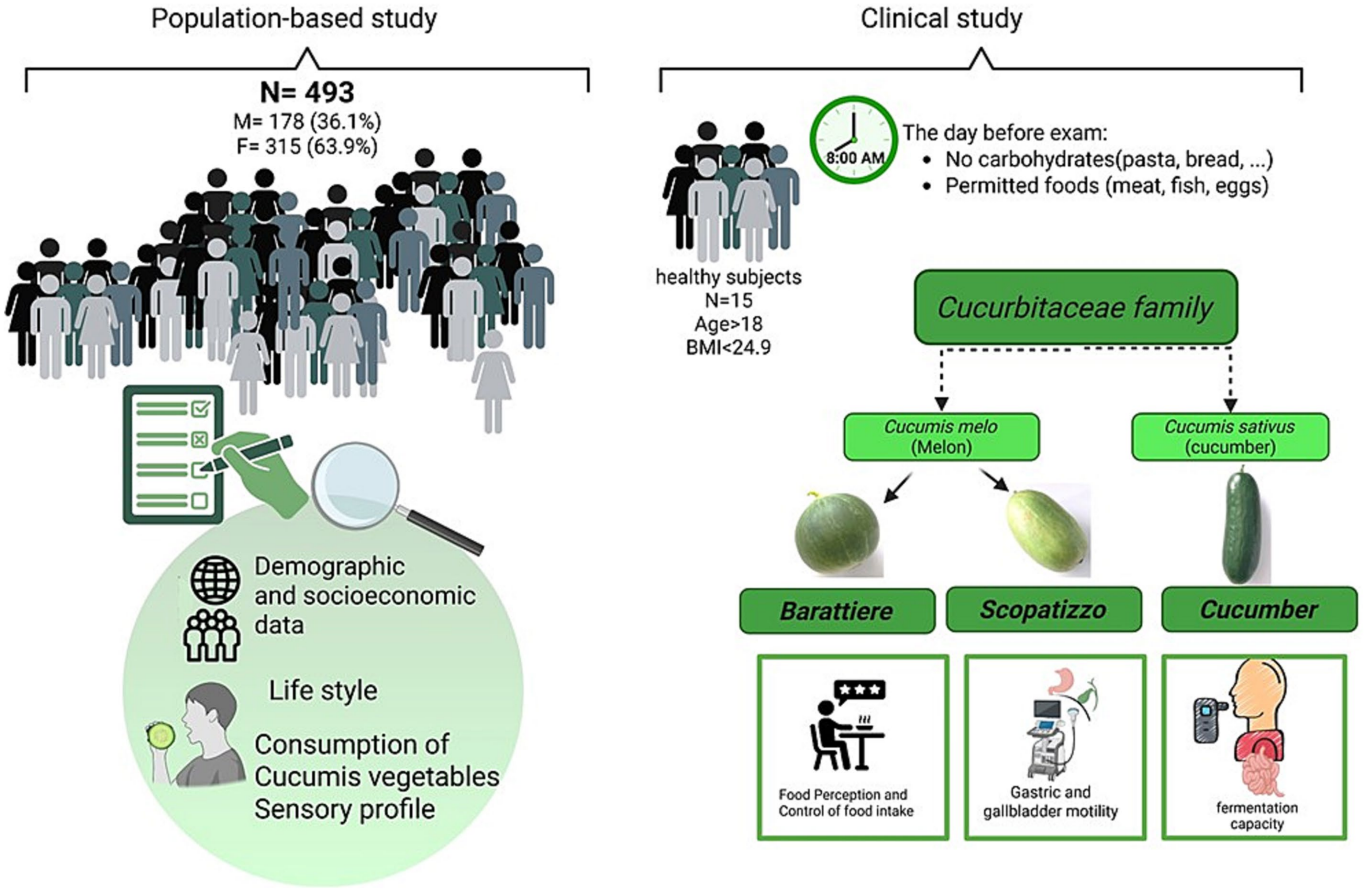
Study design.

Figure 1 shows the three vegetable varieties included in this study (Barattiere, Scopatizzo, and cucumber). The protocol included a population-based study and a specific clinical study (Figure 2).

### 2.1 Population-based study

#### 2.1.1 Survey

A cross-sectional population-based survey was conducted to assess demographic, socioeconomic, and sensory preference characteristics related to the consumption of the three *Cucumis* fruits, i.e., BAR, SCO, and CUC. The study targeted a representative sample of individuals across different age groups, educational backgrounds, geographic locations, and occupational statuses. The survey was conducted using an online questionnaire and in-person interviews to capture a diverse population.

#### 2.1.2 Questionnaire

We used a tailored, anonymous, web-based questionnaire (Google Form; Supplementary Text 1), originally developed in English and subsequently translated into Italian, following validation procedures consistent with our previous research (17, 18).

The structured questionnaire was developed to evaluate consumer preferences and the perceived digestibility of three types of *Cucumis* fruits. This tool was administered as part of a broader clinical study conducted at the Policlinico of Bari, within the framework of the ONFoods project (PNRR – Mission 4, Component 2, Investment 1.3 – Call No. 341 of March 15, 2022, funded by the European Union – NextGenerationEU).

**Figure 3:**
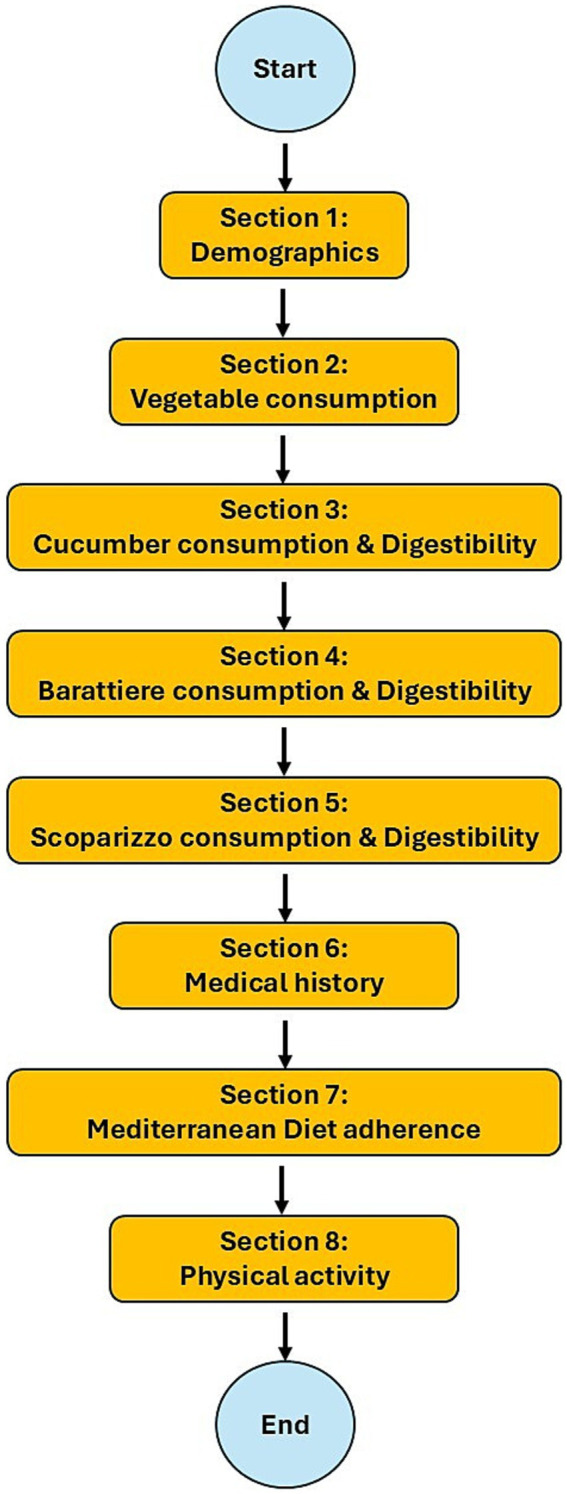
Overview of survey domains and variables assessed in the study. The questionnaire included eight sections: (1) demographics (gender, age, education level, province and area of residence, economic status, occupation); (2) vegetable consumption (frequency, awareness of fruit and vegetable intake, adherence to seasonal consumption); (3) cucumber (CUC) consumption and digestibility, including knowledge, habitual purchase, sensory evaluation (sweetness, juiciness, savoriness, crunchiness, bitterness, aromatic intensity; 5-point Likert scale), and post-consumption digestive symptoms (Likert scale from “absolutely yes” to “absolutely no,” plus open-ended responses, categorized into maldigestion or occasional discomfort); (4) Barattiere (BAR) consumption and digestibility, with the same sensory and digestive evaluation framework; (5) Scopatizzo (SCO) consumption and digestibility, with the same sensory and digestive evaluation framework; (6) medical history (alcohol and tobacco use, food allergies/intolerances, diagnosed medical conditions, current medications); (7) adherence to the Mediterranean diet, assessed by a Mediterranean adherence score (52); and (8) physical activity during the previous 7 days, categorized by intensity (moderate vs. vigorous) and setting (work, transportation, leisure) according to IPAQ guidelines (https://www.physio-pedia.com/images/c/c7/Quidelines_for_interpreting_the_IPAQ.pdf) (53).

The questionnaire comprised eight sections, designed to be completed in approximately 10 min, and collected different information, as shown in the following scheme (Figure 3).

Sampling was conducted through simple random methods to ensure representativeness and reduce selection bias. The questionnaire was disseminated via social media platforms (e.g., WhatsApp, Email, Facebook) and through face-to-face interviews for individuals without internet access, thereby supporting the external validity and inclusivity of the study.

All responses were collected anonymously and processed in accordance with the General Data Protection Regulation (GDPR, EU Regulation 2016/679).

### 2.2 Clinical protocol

#### 2.2.1 Subjects

A total of 15 healthy adult lean subjects were enrolled in the study (8 females, 7 males, ≥18 years old, mean age 31.9 ± SEM 2.6 years, BMI range 18.5–24.9 kg/m^2^ mean 22.2 ± 0.6 kg/m^2^). Subjects underwent a full medical history and physical exam to rule out functional or organic diseases. Exclusion criteria included a history of current or prior gastrointestinal diseases (e.g., inflammatory, neoplastic diseases, gastrointestinal surgery, irritable bowel syndrome, functional constipation), pregnancy, recent use of medications that could alter sensory perception or gastrointestinal motility, and probiotics, symbiotic, postbiotic or antibiotics intake in the last 3 months.

This was a real-life observational study without specific dietary restrictions regarding *Cucumis* vegetable consumption. All participants were fasting overnight and instructed to avoid carbohydrate intake after lunch on the previous day. A 3-day dietary questionnaire (19) was administered before the assessments, and review of these data confirmed that none of the 15 subjects reported Scopatizzo or Barattiere consumption during the week prior to examination, minimizing the likelihood of dietary influence on the study outcomes. All participants had prior experience in similar controlled nutrition studies and were trained to comply with the study protocol. The clinical study was designed as a randomized crossover trial in which each participant consumed all three *Cucumis* varieties (BAR, SCO, and CUC) on separate test days. This within-subject design increased statistical power by allowing each individual to act as their own control, thereby reducing inter-individual variability and amplifying the number of observations obtained from the cohort.

On three randomly selected days and at least 1 week apart, each subject underwent testing of sensory response, control of food intake, GI symptoms and GI motility studies after ingesting 80 g of BAR, SCO, or CUC. The test started at 8 a.m., and all subjects had to be fasting for at least 12 h before.

Ensuring accordance with The Code of Ethics of the World Medical Association (Declaration of Helsinki), the study received approval from the Local Ethics Committee (study 1,572/CEL Prot. n 167, approved on 5th March 2024), and all subjects signed the informed consent.

#### 2.2.2 Vegetables

The BAR and SCO were purchased from Miss Freschezza company (Monopoli, Apulia, Italy), while CUC (cv. Baby star RZ F1) were purchased from Lapietra company (Monopoli, Apulia, Italy). Freshly harvested fruits were refrigerated and then delivered to the Clinica Medica “A. Murri,” Policlinico Hospital, Bari, Italy. Before administration, all fruits were washed, blotted dry with paper towels and cut to obtain edible portions comparable in size. A part of the samples was used for the proximate analysis, which was carried out using AOAC methods (20) as follows: ashes were determined by muffle furnace according to AOAC method 923.03; proteins content (N x 6.25) was determined by Kjeldahl nitrogen, according to the AOAC method 955.04; fat content was determined by Soxhlet extraction, according to the AOAC method 920.39; dietary fiber content was determined by the enzymatic-gravimetric procedure, according to the AOAC method 991.43; and total carbohydrates were calculated by the difference of protein, lipid, and ash on the dry matter basis. Results of the proximate analysis are reported in Table 1.

**Table 1:** Nutritional value of 80 g of the three tested cucumber varieties.

	Cucumber	‘Barattiere’	‘Scopatizzo’
Calories (kcal)	12.8	10.4	8.8
Protein (g)	0.56	0.64	0.56
Lipids (g)	0.14	0.16	0.08
Carbohydrate (g)	1.44	1.6	1.44
Fiber (g)	0.64	0.24	0.16

Kcal, kilocalories; g, grams.

#### 2.2.3 Sensory studies, control of food intake, and GI symptoms

This part of the clinical study preceded the motility study and consisted of the ingestion of the three varieties of *Cucumis* fruits on different days. Examinators and most of the enrolled subjects were experienced, since they had joined previous similar studies on food perception and gastrointestinal motility, thus achieving a foundational understanding of sensory evaluation by semiquantitative scales for sensory perception (16, 19). No additional training or calibration sessions were conducted.

The visual Analogue Scales (VAS) were used to quantify different domains of perception according to previous studies of our group (21). The VAS consisted of a 100 mm horizontal line ranging from 0, i.e., no perception, to 100, i.e., max perception. Subjects had to mark the horizontal line at each time point, and the value in mm was recorded for each subject, scoring each domain and type of vegetable. Domains of perception consisted of five categories and specific subcategories, i.e., odor, taste, and chewing.

The VAS was also employed to quantify both homeostatic and hedonic (rewarding) aspects of food intake regulation, as well as GI symptoms. Homeostatic control was assessed using hunger and satiety ratings, while reward-related responses were evaluated through wanting, liking, and learning scores. Additionally, GI symptoms (i.e., nausea, fullness, epigastric pain, and meteorism) were recorded. Each participant completed the VAS questionnaires at baseline (before the test meal) and at multiple time points over a 2-h period following the consumption of the test meal. The time-dependent changes in VAS scores were plotted for each parameter. The area under the VAS curve (AUC) was calculated to quantify the overall response over time for each domain. To minimize bias and enhance the reliability of sensory evaluation, standardized testing conditions were maintained for all three tested vegetables.

#### 2.2.4 Motility studies

All enrolled subjects underwent a GI motility study by combined measurements of gastric, gallbladder emptying, and orocecal transit time during 2 h following the ingestion of the three *Cucumis* fruits.

For the stomach and gallbladder kinetics, time-dependent changes of fasting postprandial antral areas (cm^2^) and gallbladder volumes (mL) were measured simultaneously by ultrasound scans using a Sonoscape E2 Ultrasound machine equipped with a convex probe (3.4–5.0 Mhz) to previously validated techniques (22–24). Transabdominal ultrasonography is a non-invasive, reliable technique used to assess gastric and gallbladder motility. Using a standard ultrasound probe, serial measurements of the antral cross-sectional area also provide an indicator of gastric emptying, and sagittal and transversal scans of the gallbladder indicate gallbladder volume. Antral area and gallbladder volume were measured in the fasting subjects at baseline (−5 min), after meal ingestion (0 min), and every 15 min until 120 min. To ensure accuracy, these measurements were repeated at least 2 times at each time point to confirm the reliability. Gastric emptying was expressed as time-dependent changes of absolute (cm^2^) and percentage (%) antral areas. Further indices included the Area Under the emptying Curve (AUC, cm^2^ × 120 min) and the half-emptying time (T_1/2_, min). The T1/2 was calculated from linear regression analysis of the descending part of the emptying curves and expressed as “true” or estimated T_1/2_, looking at the intercept between the regression line and the horizontal line corresponding to the 50% decrease of antral area.

Gallbladder emptying indices were obtained from gallbladder volumes expressed as mL and %. The fasting volume was the gallbladder volume measured following at least 8 h of fasting. The minimum postprandial volume (mL and %) after meal ingestion was the residual volume. Additional indices included the AUC (mL × 120 min), the rate of gallbladder emptying and refilling (mL/min), the volume of ejected bile (expressed as mL and % of basal), and the half-emptying time (T_1/2_). The T_1/2_ was calculated by linear regression analysis from the descending part of the emptying curves and expressed as “true” or “estimated” T_1/2_, looking at the intercept between the regression line and the horizontal line corresponding to the 50% decrease in gallbladder volume. Gallbladder time to residual volume was the time required to reach the postprandial residual volume.

The colonic fermentation capacity and the orocecal transit time (OCTT) were measured by the H_2_-breath test following guidelines (16). In detail, breath samples were systematically collected to monitor changes associated with lactulose fermentation and H_2_ production. The collection process began with a baseline sample taken immediately before meal ingestion. Following the meal, additional breath samples were collected at regular intervals of 10 min, continuing until 120 min post-ingestion. This schedule provided a comprehensive temporal profile of H_2_ production. To ensure the reliability and reproducibility of the measurements, each breath sample was analyzed twice. If the results from the two measurements exhibited significant variability beyond a pre-defined threshold, a third measurement was conducted. The final recorded value for each time point was the average of all valid measurements taken (two or three, depending on the consistency of the initial pair).

Time-dependent changes of H_2_ in expired breath were studied using a pre-calibrated, portable hydrogen-sensitive electrochemical device (EC60Gastrolyzer; Bedfont Scientific, Medford, NJ, United States) (25). Results were expressed as H2 excretion in parts per million (device’s accuracy ±2 ppm). The OCTT (min) was defined as a rise of 10 ppm above the baseline on two consecutive measurements. This test was also considered a measure of the fermentative capacity of the tested meals. Smoking was not allowed during the tests.

### 2.3 Statistical analysis

Data were expressed as means with standard error of mean or as percentages. The normal distribution of data was assessed by Shapiro–Wilk’s test. For continuous variables, the analysis of variance was carried out by ANOVA followed by a multiple comparison test using NCSS 2023 Statistical Software (2023) (NCSS, LLC. Kaysville, Utah, United States).^2^ For the clinical studies, the sample size of 15 participants meets the minimum requirement to achieve a statistical power of 0.80 for detecting a medium effect size (Cohen’s *f* = 0.25) at an alpha level of 0.05 in a repeated-measures design, as estimated using R software. Although the overall number of subjects was modest, each participant underwent repeated assessments with three different *Cucumis* vegetables at three time points. This repeated-measures approach improves sensitivity by reducing inter-subject variability and increasing the total number of observations, thereby enhancing the ability to detect meaningful differences among conditions. A *p*-value of <0.05 was considered statistically significant, corresponding to a 95% confidence level.

## 3 Results

### 3.1 Population-based study

#### 3.1.1 General characteristics of participants

Complete questionnaires were returned from 493 participants. The demographic and socioeconomic characteristics of the population are depicted in Table 2.

**Table 2:** Demographic and socioeconomic characteristics of the study population (*N* = 493).

Categories	*N* (%)
Male	178 (36.1)
Female	315 (63.9)
Age group (years)	
18–35	122 (24.8)
36–64	324 (65.7)
>64	47 (9.5)
Education level	
Primary School	10 (2.0)
High school	101 (20.5)
Bachelor’s degree	47 (9.5)
Master’s degree	168 (34.1)
Doctorate or Specialized Higher Education	167 (33.9)
Geographical distribution	
Apulia	434 (88.0)
Piedmont	12 (2.4)
Lazio	11 (2.2)
Basilicata	9 (1.8)
Other	27 (5.5)
Residential environment	
Urban	461 (93.5)
Rural	32 (6.5)
Self-reported economic status	
Low	18 (3.7)
Mild	33 (6.7)
Moderate	240 (48.7)
Good	162 (32.9)
Very good	40 (8.1)
Occupational status	
Public Sector Employee	333 (67.6)
Self-Employed Professional	37 (7.5)
Students	48 (9.7)
Retired	22 (4.5)
Unemployed	15 (3.0)
Others	
Comorbidities and lifestyle	
Type 2 diabetes	16 (3.2)
Dyslipidemia	13 (2.6)
Hypertension	27 (5.5)
Gastrointestinal disorders	19 (3.9)
Alcohol consumers	323 (65.5)
Smokers	90 (18.3)

N, number of subjects.

There was a higher prevalence of females (63.9%). The age groups included 24.8% aged 18–35 years, 65.7% aged 36–64 years, and 9.5% over 64 years. Most participants were from Apulia (88.0%) and were urban residents (93.5%). Educational levels varied, with 20.5% having a high school diploma, 9.5% holding an undergraduate degree, 34.1% possessing a master’s degree, and 33.9% achieving a doctorate or specialized higher education. Economic status was self-reported as low (3.7%), mild (6.7%), moderate (48.7%), good (32.9%), or very good (8.1%). Occupational distribution included 67.6% public sector employees, 7.5% self-employed professionals, 9.7% students, 4.5% retirees, and 3.0% unemployed individuals. Few subjects reported type 2 diabetes (3.2%), dyslipidemia, (2.6%), hypertension (5.5%), and GI disorders (3.9%). Lifestyle data showed that 65.5% consumed alcohol, and 18.3% were smokers.

#### 3.1.2 Dietary habits

Table 3 depicts the dietary intake patterns among participants. The majority consumed 100–150 g/day of fruits (39.2%) and 100–250 g/day of vegetables (39.0%). Most participants had 70–140 g/week of legumes (47.3%) and consumed less than 130 g/day of cereals (60.5%). Regarding protein sources, 45.2% consumed 100–250 g/week of fish, while 56.0% consumed more than 120 g/day of meat. Dairy consumption was high, with 77.5% consuming more than 270 g/day. Alcohol intake was low, with 92.3% drinking less than one drink per day (1 drink equaling ~12 g of alcohol and 84 kcal). Olive oil as a dietary staple was consumed regularly by 91.9% individuals. The mean Mediterranean Diet (MD) adherence score was 11.0 ± 0.1, with 41.6% classified as high adherence (score 13–18).

**Table 3:** Dietary intake patterns and adherence to the Mediterranean diet among participants.

Food categories	*N* (%)
Fruits	
<100 g/day	166 (33.6)
100–150 g/day	193 (39.2)
>150 g/day	134 (27.2)
Vegetables	
<100 g/day	132 (26.8)
100–250 g/day	192 (39.0)
>250 g/day	169 (34.2)
Legumes	
<70 g/week	158 (32.0)
70–140 g/week	233 (47.3)
>140 g/week	102 (20.7)
Cereals	
<130 g/day	298 (60.5)
130–200 g/day	155 (31.4)
>200 g/day	40 (8.1)
Fish	
<100 g/week	193 (39.2)
100–250 g/week	223 (45.2)
>250 g/week	77 (15.6)
Meat	
<80 g/day	67 (13.6)
80–120 g/day	150 (30.4)
>120 g/day	276 (56.0)
Dairy products	
<180 g/day	20 (4.0)
180–270 g/day	91 (18.5)
>270 g/day	382 (77.5)
Alcohol	
<1 drink/day	493 (92.3)
1–2 drinks/day	38 (7.3)
>2 drinks day	2 (0.4)
Olive oil	
Occasional	1 (0.2)
Frequently	39 (7.9)
Regularly	451 (91.9)
MD score (0–18) (mean + SEM)	11.0 ± 0.1
Low (0–6)	24 (4.9)
Moderate (7–12)	264 (53.5)
High (13–18)	205 (41.6)

MD score is expressed as mean± SEM. g, Grams; MD, Mediterranean diet; N, number of participants; SEM, standard error of the mean.

#### 3.1.3 Consumption prevalence, digestive tolerance, and sensory profile of *Cucumis* fruits

**Figure 4:**
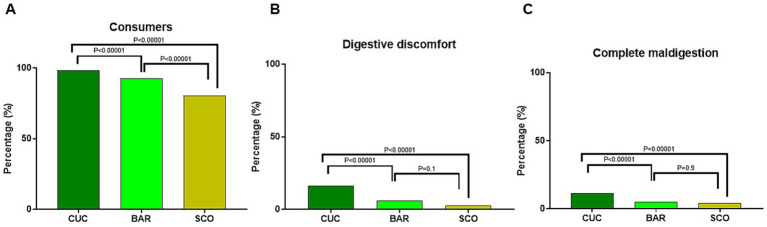
Reported consumption prevalence **(A)**, digestive discomfort **(B)**, and complete maldigestion **(C)** of CUC, BAR, and SCO among the participants. Statistical analysis was performed using the chi-square test.

The consumption rates of the 3 vegetables were high among subjects, with 98.4% of participants consuming CUC, 92.7% consuming BAR, and 79.5% consuming SCO (*p* < 0.00001) (Figure 4A). Reported mild digestive issues (i.e., discomfort, mild indigestion) and complete maldigestion including symptoms such as postprandial fullness, abdominal distension, and flatulence were low but higher (*p* < 0.00001) in CUC consumers (16.3 and 11.5%, respectively) than in BAR consumers (4.6 and 4.2%) and SCO consumers (2.6 and 4.1%) (Figures 4B,C).

**Figure 5:**
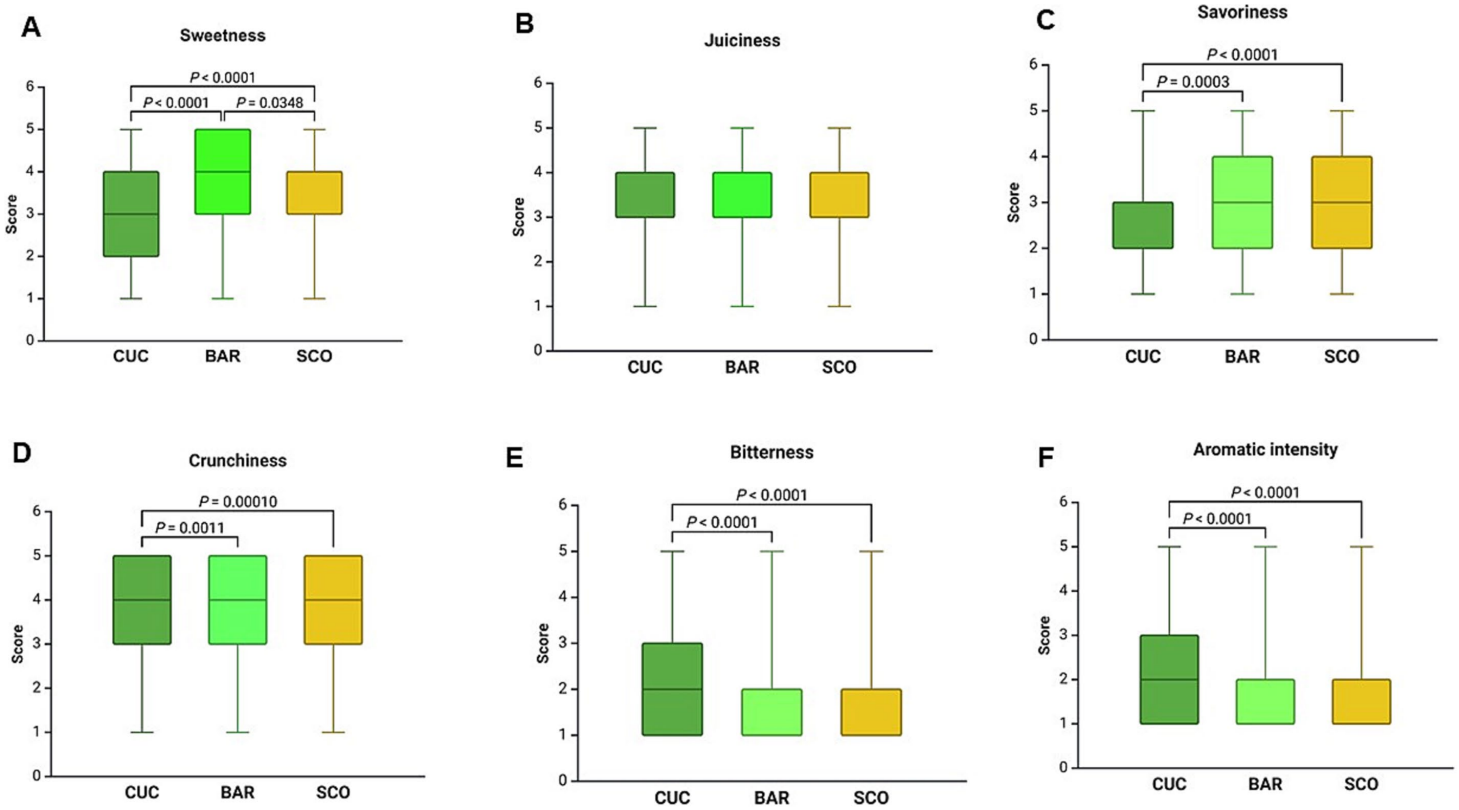
Sensory profiles of CUC, BAR, and SCO expressed as violin graphs of scores vs. *Cucumis* fruit. **(A)** Sweetness, **(B)** juiciness, **(C)** savoriness, **(D)** crunchiness, **(E)** bitterness, **(F)** aromatic intensity. Data are expressed as mean ± SEM. Statistical analysis was performed using the Kruskal–Wallis test, followed by a multiple comparisons *post hoc* test to identify significant differences among the three varieties.

The results of sensory evaluations are given in Figure 5, revealing significant differences across the three *Cucumis* vegetables, except for freshness and juiciness. Sweetness differed significantly among all three foods (*p* < 0.00001), with BAR scoring the highest (3.77 ± 0.05). Crunchiness was significantly higher in SCO (4.01 ± 0.05) than CUC (3.76 ± 0.05) (*p* = 0.002). Bitterness was lowest in BAR (1.51 ± 0.04) and highest in CUC (2.20 ± 0.05, *p* < 0.00001). Aromatic intensity was significantly higher in SCO compared to CUC and BAR (*p* < 0.00001).

### 3.2 Clinical study

#### 3.2.1 Food perception and control of food intake

Table 4 depicts the differences in eating behavior, sensory perception, and GI symptoms across CUC, BAR, and SCO. Chewing count (for eating 80 g of each vegetable) differed significantly (*p* = 0.02), with SCO having the highest number of chews (18.0 ± 1.7) and CUC the lowest (12.4 ± 1.6). Sensory profile and GI symptoms did not show significant differences, except for a higher trend in bitter taste perception (*p* = 0.06) in CUC and particle breakdown in BAR (*p* = 0.06).

**Table 4:** Food intake control, sensory profile, and gastrointestinal symptoms in response to 80 g of CUC, BAR, and SCO.

	CUC (*N* = 15)	BAR (*N* = 15)	SCO (*N* = 15)	*p*
Eating time (min)	2.4 ± 0.2	2.1 ± 0.2	2.7 ± 0.2	0.07
Number of chews	12.4 ± 1.6*	13.0 ± 1.0	18.0 ± 1.7*	0.02
Sensory profile VAS (0–100 mm)				
Odor intensity	50.5 ± 7.4	52.0 ± 8.7	40.0 ± 6.1	0.47
Strong taste	34.0 ± 6.0	43.0 ± 9.9	27.5 ± 5.7	0.31
Bitter taste	24.0 ± 9.2	1.0 ± 1.0	2.0 ± 1.3	0.06
Sour taste	4.0 ± 2.2	1.0 ± 1.0	3.0 ± 2.1	0.52
After taste	35.0 ± 7.8	39.0 ± 9.8	33.0 ± 8.3	0.81
Particle breakdown	65.5 ± 4.6	80.5 ± 5.9	77.0 ± 2.0	0.06
Hardness	55.0 ± 5.8	43.0 ± 5.4	49.0 ± 5.0	0.31
GI Symptoms (AUC VAS*120 min)				
Fullness	1,056 ± 410	461 ± 191	493 ± 249	0.41
Epigastralgia	0 ± 0	0 ± 0	0 ± 0	–
Nausea	44.8 ± 44.8	10 ± 10	44.8 ± 44.8	0.4
Meteorism	218.5 ± 180.3	180 ± 180	89.8 ± 71.0	0.82

Data are expressed as mean ± SEM. AUC, area under the curve; GI, gastrointestinal; N, Number of subjects; VAS, visual analog scale. Differences were tested by the Kruskal–Wallis multiple-comparison *Z*-value test and Dunn’s test for multiple comparisons. Similar symbols indicate a significant difference.

**Figure 6:**
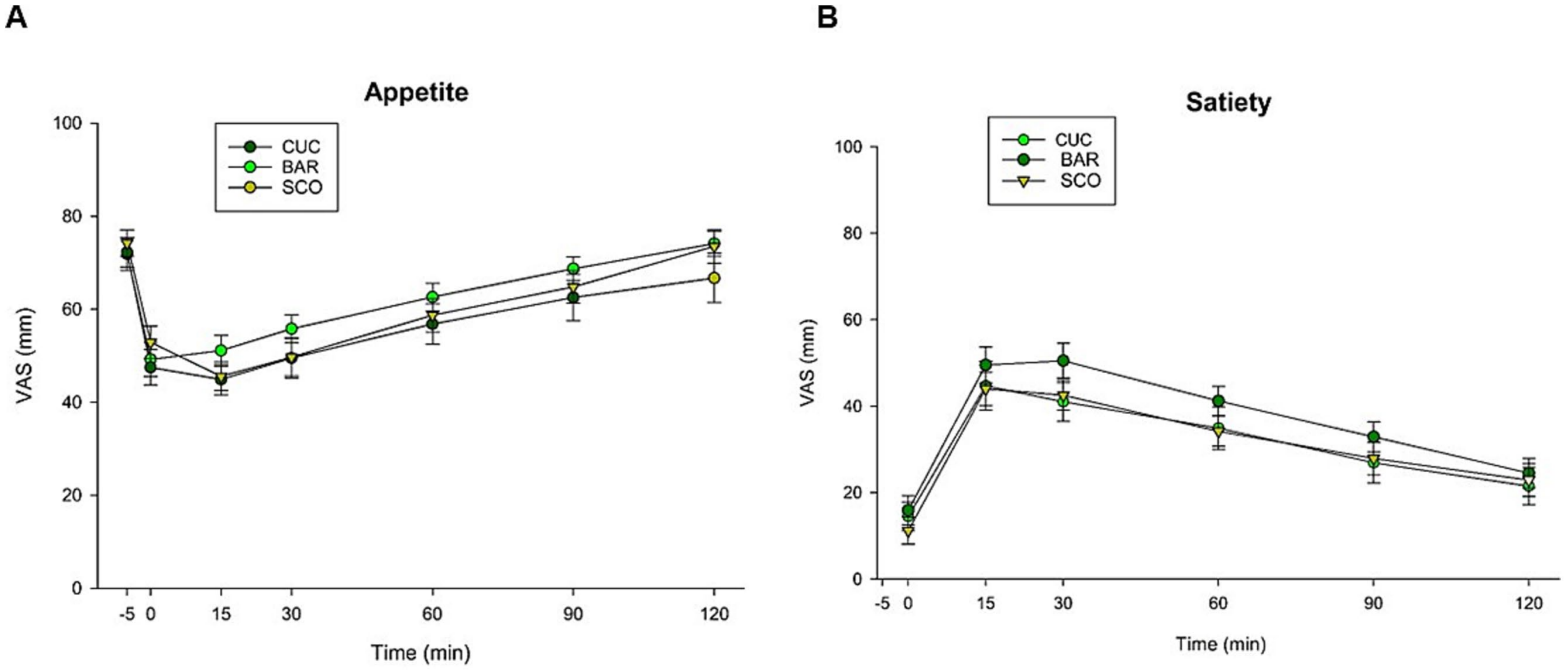
Time-dependent changes in **(A)** appetite and **(B)** satiety after consuming the test meal CUC, BAR, and SCO.

Food intake control measures (VAS) showed no significant differences for homeostatic food intake control (appetite and satiety) across the three fruits (Figure 6). In detail, Figure 6A shows the time-dependent change in appetite following consumption of BAR, SCO, and CUC. Appetite decreased from a baseline (−5 min) of VAS 72–74 to VAS 47–53 immediately after consumption (0 min), then gradually increased to VAS 66–74 over the following 2 h. Satiety (Figure 6B) increased from a baseline (−5 min) of VAS 11–16 to VAS 40–50 immediately after eating (0 min), followed by a gradual decline over the next 2 h to VAS 13–15. While BAR showed a trend toward a higher satiety response compared to SCO and CUC, the differences between the groups were generally comparable.

**Figure 7:**
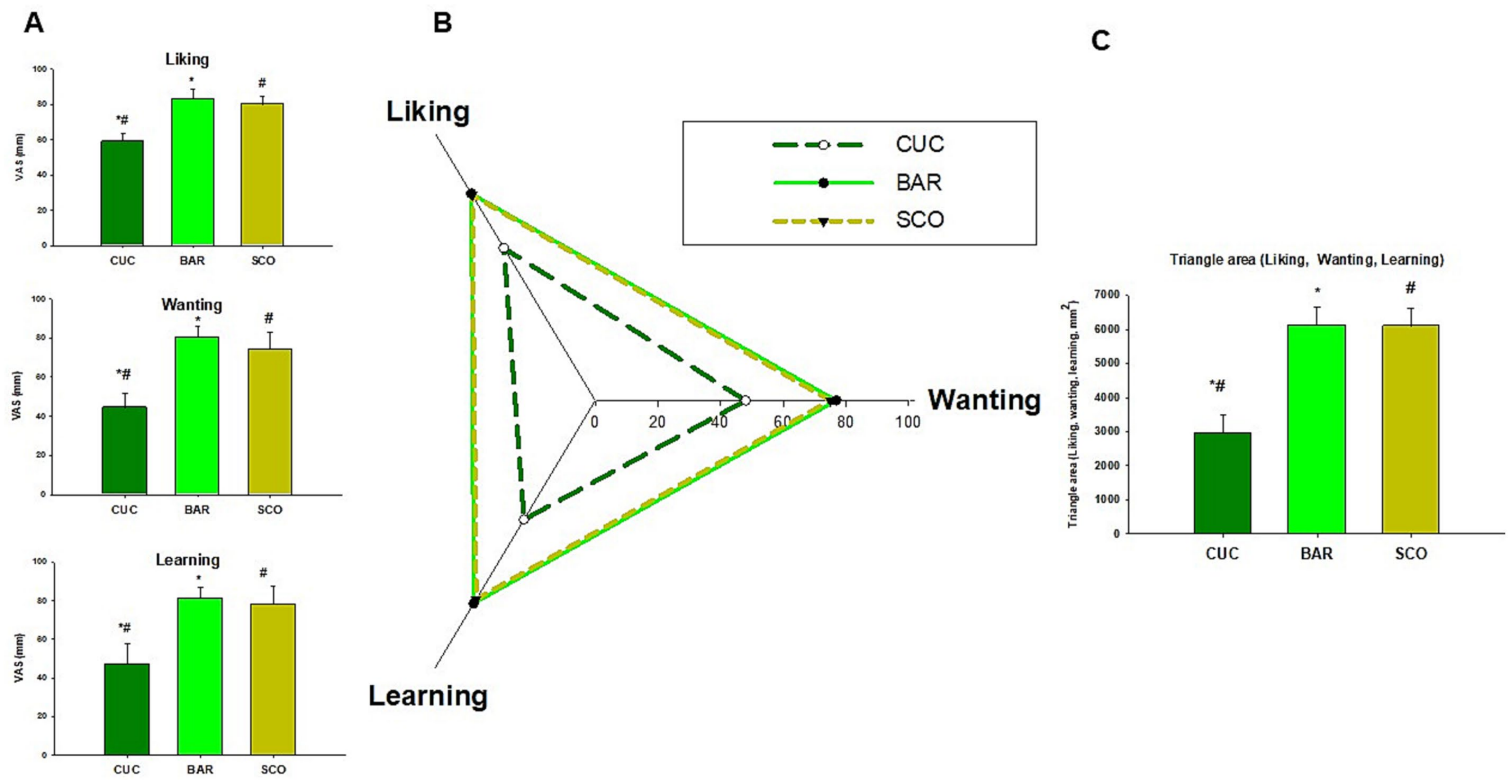
Data on rewarding food intake control (liking, wanting, and learning) in response to CUC, BAR, and SCO consumption. **(A)** VAS (0–100 mm); **(B)** radar plot; **(C)** triangle area from radar plot. With bars, data are expressed as mean ± SEM. Statistical analysis was performed using the Kruskal–Wallis test, followed by a multiple comparisons *post hoc* test to identify significant differences among the three varieties. Similar symbols (*, #) indicate a significant difference.

The results concerning the rewarding response to food intake are summarized in Figures 7A–C. Significant differences existed among groups, with CUC scoring lower in liking (59.0 ± 4.3), wanting (44.5 ± 7.5), and learning (47.0 ± 10.4) compared to BAR and SCO (*p* < 0.02) (Figure 7A). Results are visually emphasized in the radar plot, which depicts the triangles constructed by scoring liking, wanting, and learning (Figure 7B). The areas from each triangle are noticeably smaller for CUC, highlighting its reduced reward-related response, compared to BAR and SCO (Figure 7C).

#### 3.2.2 Gastric motility

**Figure 8:**
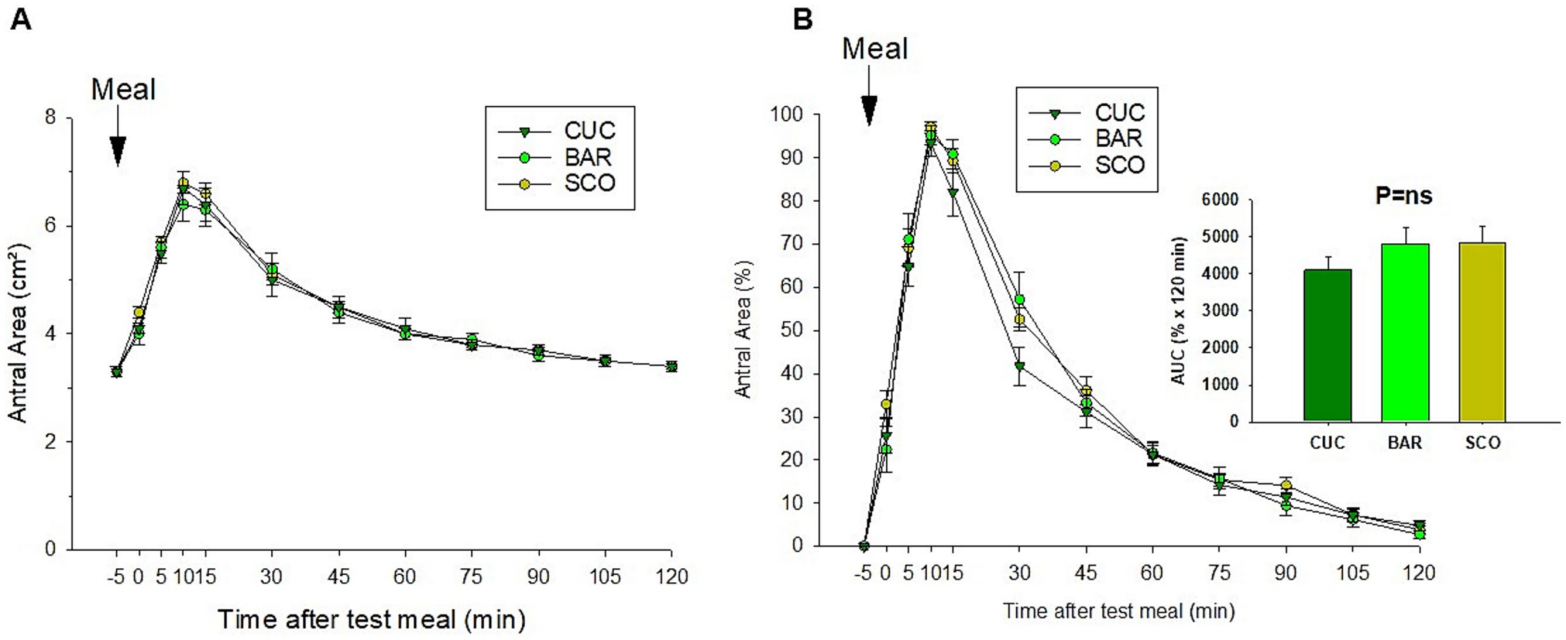
Time-dependent changes in stomach antral are (**A** in cm^2^ and **B** in percentage of baseline area) in response to CUC, BAR, and SCO test meals. Differences were tested by Kruskal–Wallis multiple-comparison *Z*-value test and Dunn’s test for multiple comparisons.

Table 5 reports gastric motility responses to BAR, SCO, and CUC. No significant differences were observed across the tested meals. Basal, maximal, and minimal gastric areas were comparable, with maximal area ranging from 6.7 ± 0.34 cm^2^ (BAR) to 7.0 ± 2.6 cm^2^ (SCO). Half-emptying time (tT_1/2_) was similar across meals, with CUC at 36.6 ± 4.65 min and SCO at 33.0 ± 1.46 min (*p* = 0.6). Figure 7 shows comparable patterns of both absolute (Figure 8A) and normalized (Figure 8B) time-dependent changes in the stomach antrum area, with no significant variation of AUC indicating similar gastric motility responses among the three test meals.

**Table 5:** Gastric motility in response to CUC, BAR, and SCO test meals.

	CUC (*N* = 15)	BAR (*N* = 15)	SCO (*N* = 15)	*P*
Basal area (cm^2^)	3.2 ± 0.11	3.3 ± 0.13	3.2 ± 0.08	0.9
Maximal area (cm^2^)	6.8 ± 0.33	6.7 ± 0.34	7.0 ± 2.6	0.6
Minimal area (cm^2^)	3.4 ± 0.12	3.4 ± 0.13	3.3 ± 0.06	0.9
Minimal area (%)	5.8 ± 1.45	4.9 ± 1.32	4.6 ± 0.98	0.9
tT_1/2_ (min)	36.6 ± 4.65	36.9 ± 2.34	33.0 ± 1.46	0.6
eT_1/2_ (min)	–	–	–	–
AUC (cm^2^ x 120 min)	536.0 ± 26.1	555.4 ± 23.4	544.3 ± 9.0	0.8
AUC (% x 120 min)	3,605 ± 316.6	4,069 ± 316.0	3,970 ± 209.0	0.4

Data are expressed as mean ± SEM. AUC, area under curve; eT1/2, estimated half-emptying time; N, Number of subjects; tT1/2, true half-emptying time. Differences tested by Kruskal–Wallis multiple-comparison *Z*-value test and Dunn’s test for multiple comparisons.

#### 3.2.3 Gallbladder motility

**Figure 9:**
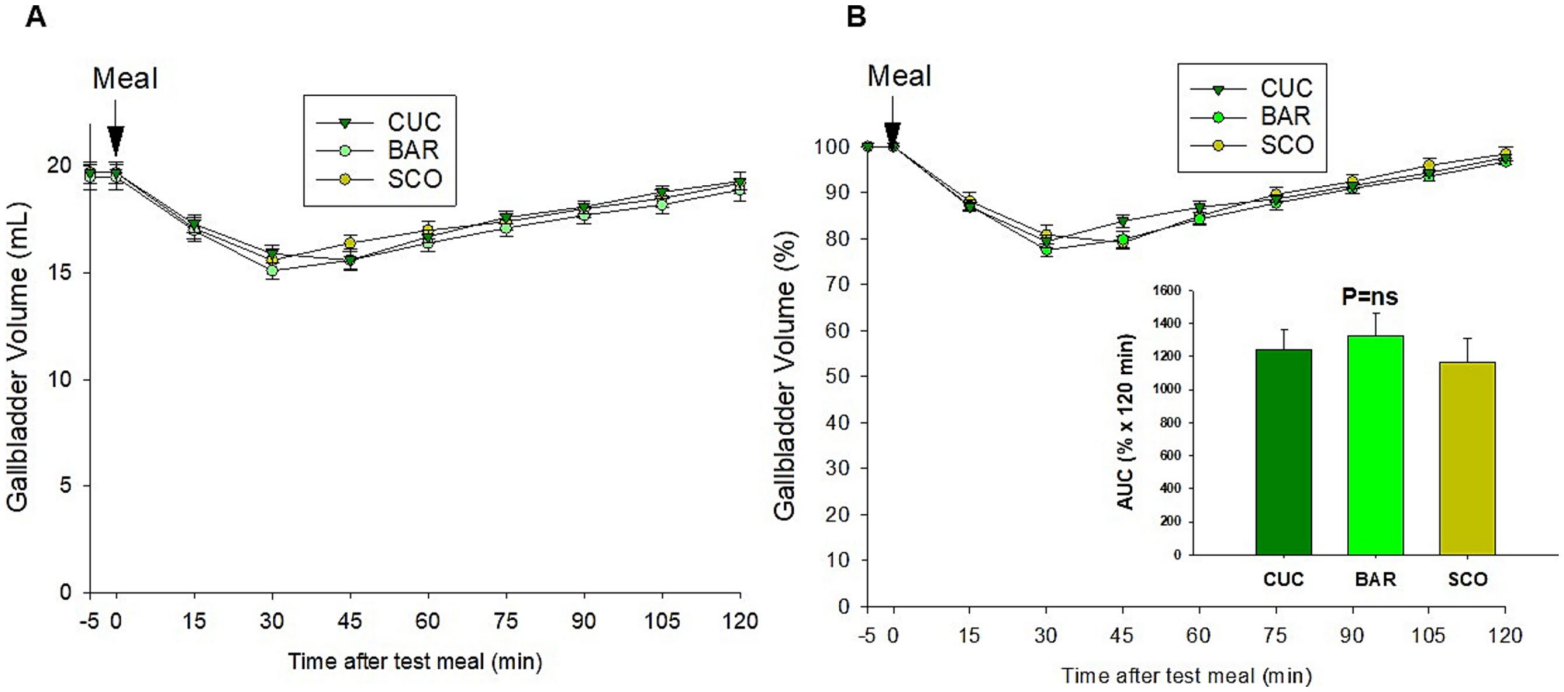
Time-dependent change in gallbladder volume (**A** in mL and **B** in percentage of fasting volume) in response to CUC, BAR, and SCO test meals.

Table 6 shows the gallbladder motility patterns in response to CUC, BAR, and SCO. Fasting and residual volumes were similar across the tested fruits. Overall, the entity of relative gallbladder contraction averaged 20% with the three vegetables. However, significant differences were observed in time to residual volume (*p* = 0.02), with CUC showing the shortest time (28.5 ± 2.7 min) and SCO the longest (39.0 ± 2.5 min). Gallbladder “true” half-emptying tT_1/2_ and “estimated” eT_1/2_ differed significantly (*p* < 0.01), with SCO showing the slowest emptying (tT_1/2_ = 23.7 ± 2.3 min, eT_1/2_ = 94.9 ± 4.3 min). The refilling rate also varied (*p* = 0.01), with CUC showing the lowest rate (0.03 ± 0.002 mL/min) and SCO the highest (0.05 ± 0.005 mL/min). These findings indicate minor differences in gallbladder motility depending on the test meal; however, the time-dependent changes in gallbladder volume (Figure 9) showed comparable patterns with no significant difference in AUC.

**Table 6:** Gallbladder motility parameters in response to CUC, BAR, and SCO test meals.

	CUC (*N* = 15)	BAR (*N* = 15)	SCO (*N* = 15)	*p*
Fasting volume (mL)	19.2 ± 0.6	19.2 ± 0.7	19.3 ± 0.7	0.94
Residual volume (mL)	15.2 ± 0.7	15.0 ± 0.6	14.8 ± 0.4	0.95
Residual volume (%)	79.0 ± 1.7	78.3 ± 1.7	77.2 ± 1.8	0.86
Time to residual volume (min)	28.5 ± 2.7*	33.0 ± 2.0	39.0 ± 2.5*	0.02
Ejection volume (mL)	4.0 ± 0.3	4.16 ± 0.4	4.5 ± 0.5	0.95
Ejection volume (%)	21.0 ± 1.7	21.7 ± 1.7	22.8 ± 1.8	0.87
Emptying rate (mL/min)	−0.13 ± 0.01	−0.13 ± 0.01	−0.11 ± 0.007	0.49
Emptying rate (%/min)	−0.68 ± 0.06	−0.67 ± 0.05	−0.58 ± 0.04	0.33
Emptying tT_1/2_ (min)	15.0 ± 1.6#	14.0 ± 1.0*	23.7 ± 2.3*#	0.0008
Emptying eT_1/2_ (min)	69.6 ± 4.7#	78.9 ± 5.2*	94.9 ± 4.3*#	0.002
Final volume (mL)	18.6 ± 0.62	18.6 ± 0.6	18.8 ± 0.31	0.66
Final volume (%)	96.8 ± 0.79	97.0 ± 1.1	98.2 ± 2.3	0.42
Refilling rate (ml/min)	0.03 ± 0.002*#	0.04 ± 0.005*	0.05 ± 0.005#	0.01
Refilling rate (%/min)	0.16 ± 0.01*	0.22 ± 0.03	0.25 ± 0.02*	0.02
Refilling tT_1/2_ (min)	58.0 ± 4.4	68.3 ± 4.1	64.7 ± 3.3	0.32
Refilling eT_1/2_ (min)	-	-	-	-
AUC (mL x 120 min)	240.7 ± 18.2	270.3 ± 32.2	242.6 ± 55.4	0.26
AUC (% x 120 min)	1,261 ± 85.6	1,399 ± 142	1,204 ± 213	0.19

Data are expressed as mean ± SEM. AUC, area under the curve; tT1/2, true half-emptying time; min, minutes. Differences were tested by Kruskal–Wallis multiple-comparison *Z*-value test and Dunn’s test for multiple comparisons. Similar symbols indicate a significant difference.

#### 3.2.4 Fermentation capacity

**Figure 10:**
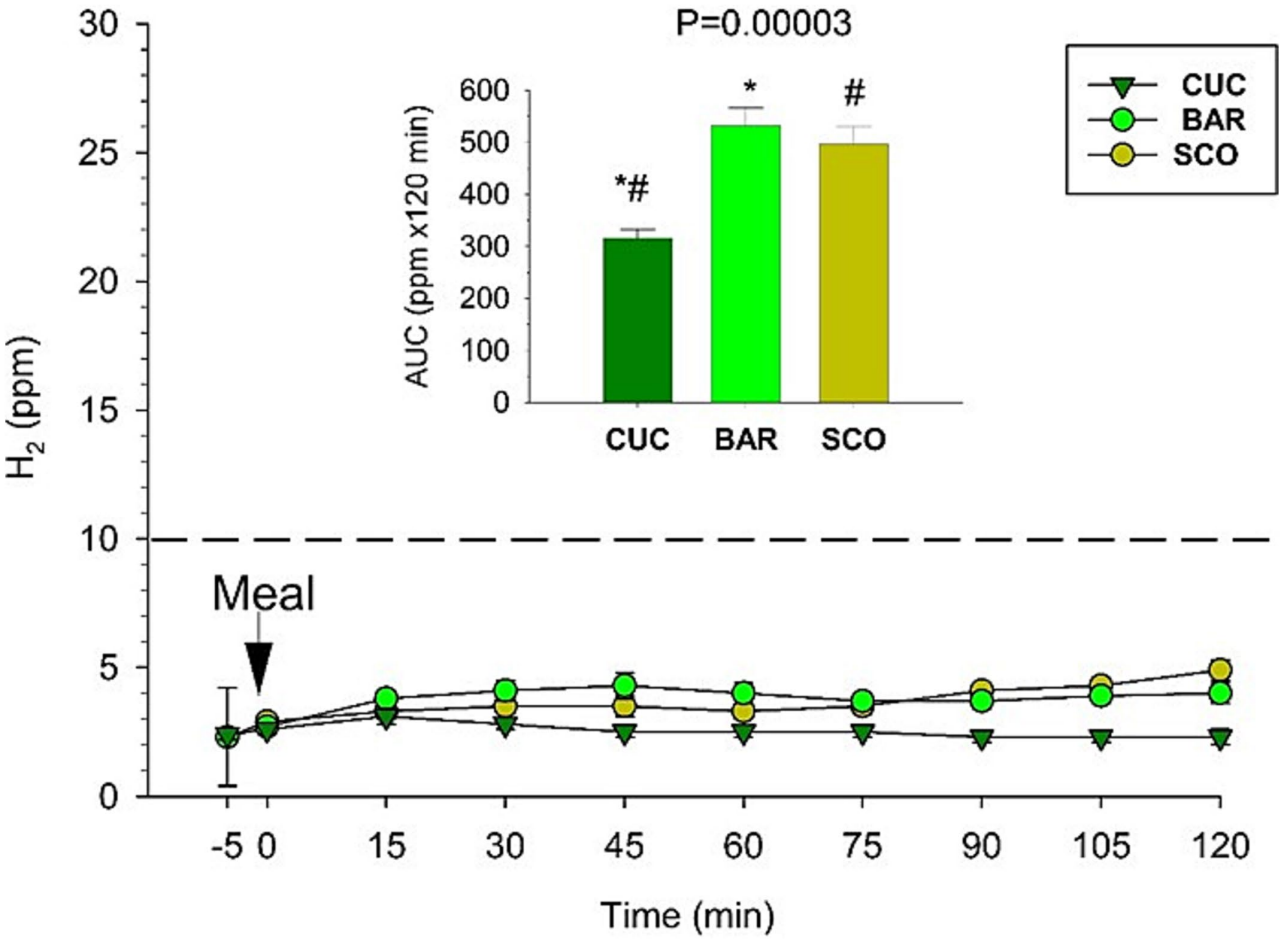
Time-dependent changes in H_2_ concentration in exhaled air in response to CUC, BAR, and SCO test meals. Differences were tested by Kruskal–Wallis multiple-comparison *Z*-value test and Dunn’s test for multiple comparisons. Similar symbols (*, #) indicate a significant difference.

Overall time-dependent changes in H₂ concentration (ppm) in exhaled air remained minimal in response to CUC, BAR, and SCO. However, BAR and SCO produced slightly but significantly higher H₂ production compared to CUC during 120 min. Due to this low response, transit time (+10 ppm from baseline) could not be determined (Figure 10).

## 4 Discussion

The novelty of this study consists of its highly translational physiological value, in that specific popular foods were extensively investigated for their consumption patterns, agreeability, and GI kinetics, to depict a comprehensive profile of foods in a “real-life” scenario.

Three *Cucumis* vegetables, which are very popular in the Apulian way of living, were investigated in population-based and controlled clinical settings.

Vegetables belonging to the *Cucumis* genus, including cucumber (*Cucumis sativus*) and various *Cucumis melo* varieties, represent a significant component of MD diets due to their high-water content, low caloric value, and richness in micronutrients and phytochemicals (26, 27). The results of our study confirm the data reported in the literature, considering that the caloric intake ranges between 8.8 and 12.8 kcal per serving (Table 1). Moreover, it is well established that most vegetables consist predominantly of water and contain low amounts of proteins, fats, and carbohydrates. The three vegetables examined in our study also show a low fiber content, with values ranging between 0.16 and 0.64 g per serving. *C. sativus* is among the most widely cultivated vegetables worldwide, particularly valued for its hydrating properties and potential health benefits (28). In recent years, consumer interest in sustainable and regionally adapted crops has attracted attention toward local cultivars, such as *Cucumis melo* landraces traditionally consumed in Southern Italy. These local varieties, including ‘Scopatizzo*’* and ‘Barattiere’, are botanically classified as immature fruits of *C. melo* but are consumed similarly to cucumbers due to their crisp texture and mild flavor (9). They are particularly appreciated in Apulia and Basilicata for their refreshing qualities, lower bitterness, and enhanced digestibility. Nutritional analyses have shown that these landraces may differ in their phytochemical profile, including lower levels of cucurbitacins and higher concentrations of bioactive compounds such as flavonoids and carotenoids, potentially offering improved gastrointestinal tolerance and health benefits (29). As such, promoting these alternative *Cucumis* varieties aligns with biodiversity conservation, cultural heritage, and consumer demand for locally sourced, functional foods.

In the population-based survey, which included a relatively large number of individuals, high consumption rates of the studied *Cucumis* vegetables were reported. CUC (98.4%) was the most frequently consumed, followed by BAR (92.7%) and SCO (79.5%). These findings reflect the widespread use of these vegetables in Apulia and are consistent with the high adherence to healthy dietary patterns, as evidenced by moderate-to-high adherence to the Mediterranean Diet (MD) in 95.1% of respondents. Dietary intake patterns also aligned with MD principles, with frequent consumption of vegetables, legumes, fish, and olive oil. However, 60.5% of participants consumed less than 130 g/day of cereals, below traditional MD recommendations. Conversely, dairy consumption was relatively high, with 77.5% exceeding 270 g/day, reflecting regional dietary preferences. Alcohol consumption was limited, with 92.3% reporting less than one drink per day, in accordance with MD guidelines. Importantly, these percentages refer exclusively to the population-based survey and not to the smaller clinical study sample, thereby avoiding confusion between the two datasets.

Several studies have shown that there is a correlation between an adequate socioeconomic level and higher adherence to the MD (30–32). This appears to be in line with our data since most participants have medium to high rates of adherence to the MD, and a medium to high level of education, as well as adequate socio-economic and employment status.

While the widespread consumption suggests a strong cultural or dietary preference, digestive tolerance varied significantly. In our population study, 16% of participants reported abdominal discomfort after consuming cucumbers, with 11% experiencing complete maldigestion, including symptoms like postprandial fullness, abdominal distension, and bloating due to excessive gas (flatulence). These findings align with existing scientific literature, which attributes such reactions primarily to cucurbitacins, bitter-tasting compounds naturally present in cucumbers and other members of the Cucurbitaceae family (33, 34). Cucurbitacins are triterpenoid compounds that impart bitterness to cucumbers (35). While typically concentrated in the leaves and stems, environmental stressors such as drought, high temperatures, and inconsistent watering can lead to their accumulation in the fruit itself (36). This increased concentration enhances bitterness and can cause gastrointestinal symptoms like nausea, stomach cramps, and diarrhea in sensitive individuals (37–39). A study (40) identified cucurbitacin C as a key bitter compound in cucumbers, with higher concentrations found at the stem end of the fruit. The threshold for detecting bitterness was noted to be less than 0.1 mg/L, indicating that even small amounts can be perceptible and potentially problematic for digestion. Thus, the digestive discomfort and “maldigestion” reported by participants in the population study can be scientifically attributed to the presence of cucurbitacins in cucumbers. Factors such as environmental stress during cultivation, individual genetic sensitivity to bitterness, and underlying digestive conditions contribute to these adverse reactions. Conversely, SCO demonstrated the lowest digestive complaints, aligning with its high sensory ratings in aromatic intensity and savoriness. In fact, sensory evaluations highlighted significant differences among the three fruits. BAR was perceived as the sweetest, while CUC was rated highest in bitterness. The increased bitterness perceived in CUC could indicate a higher concentration of cucurbitacin compared to BAR and SCO varieties. This interpretation aligns with findings from several studies that have quantified cucurbitacin C (CucC), a tetracyclic triterpenoid compound responsible for bitterness in cucumbers. Few studies (40, 41) reported CucC concentrations depending on the genotype. Bitter cucumber contains more CucC than sweet cucumber. However, it is important to note that quantitative data on cucurbitacin levels in Apulian *Cucumis* landraces is currently lacking. Therefore, while sensory observations suggest higher cucurbitacin content in CUC, further analytical studies are necessary to confirm this in local varieties.

Crunchiness was most pronounced in SCO, which aligns with its higher chewing count during the clinical study.

It is clear from several studies that the control of food intake cannot be understood only through the lens of homeostatic mechanisms, simply related to hunger and satiety. Non-homeostatic factors, such as liking, wanting and learning, play an equally important role in determining our food choices (42, 43).

Understanding these processes can pave the way to more effective strategies to promote healthier food choices by making people aware not only of the biological hunger signals, but also of the psychological and cultural influences that drive eating behavior (44). These textural and flavor attributes likely influenced food intake control measures, where CUC scored lower in liking, wanting, and learning compared to BAR and SCO. In our clinical studies, these findings were reinforced by the observation that BAR and SCO were associated with higher sensory appeal and better digestive tolerance, ultimately leading to more favorable consumer reward responses compared to CUC.

The differences in sensory perception and hedonic responses may be relevant in understanding individual food preferences and dietary choices, which are crucial for nutritional interventions aimed at improving adherence to healthy eating patterns.

Gastric motility parameters showed no significant differences across the three test meals, indicating a mild gastric dilatation and comparable gastric response and emptying rates. The lack of variation suggests that despite differences in texture and sensory attributes, these vegetables do not significantly impact gastric physiological kinetics. However, gallbladder motility exhibited distinct responses, likely due to activation of gastric afferent vagal pathways following gastric dilatation. Duodenal cholecystokinin (CCK) release by duodenal enteroendocrine I-cells in response to ingested fat and amino acids is unlikely or very limited in this context due to the poor content of both components in the 80 g of three *Cucumis* fruits (22). CUC demonstrated the shortest time to residual volume (28.5 min), while SCO had the longest (39.0 min). Furthermore, SCO exhibited the slowest emptying rate (tT_1/2_ = 23.7 min, eT_1/2_ = 94.9 min), suggesting a potential impact on bile, bile acid secretion dynamics, and enterohepatic circulation. These differences may have implications for lipid digestion, metabolic responses, and microbiota profile (45, 46), warranting further investigation into their role in postprandial lipid metabolism, short-chain fatty acid production by intestinal microbiota digesting fiber, and satiety regulation (47). This bitterness perception may also be linked to the activation of bitter taste receptors (T2Rs), which are not limited to the oral cavity but are also expressed throughout the gastrointestinal tract (48). Activation of intestinal T2Rs by bitter compounds has been associated with the stimulation of gastrointestinal motility and bile release, suggesting a possible cholecystokinetic effect (49, 50). Therefore, the observed differences in gallbladder behavior in response to cucumber consumption may be explained by a higher activation of bitter receptors due to increased cucurbitacin content.

Importantly, while vegetable-containing cucurbitacins have been associated with gastrointestinal toxicity at higher concentrations (37, 38, 51), the levels potentially present in the CUC variety tested in this study might not be sufficient to elicit overt symptoms, but could contribute to the perception of a “heavier” or more difficult digestion. This is supported by participant feedback indicating greater digestive discomfort following CUC intake compared to BAR and SCO.

Further studies analyzing the actual cucurbitacin content of these varieties could substantiate this hypothesis and clarify whether bitterness perception correlates with bioactive compound concentration and digestive response.

The results of this study reveal minimal colonic hydrogen (H₂) production in response to the ingestion of CUC, BAR, and SCO, as reflected by the low concentrations of H₂ in exhaled breath samples. Although BAR and SCO demonstrated relatively higher H₂ levels compared to CUC, the overall magnitude of H₂ production was small across all conditions. This suggests limited fermentative activity in the colon following the ingestion of these melon varieties, at least during 2 h postprandially.

Hydrogen gas in the breath is primarily produced through bacterial fermentation of undigested carbohydrates that reach the colon. The low H₂ response observed here may indicate that the digestible carbohydrate content in these melons is efficiently absorbed in the small intestine, resulting in minimal substrate availability for colonic microbiota. Alternatively, these varieties may possess a carbohydrate profile that is inherently less fermentable, either due to low fiber content or specific types of sugars (e.g., sucrose, glucose, and fructose) that are readily absorbed prior to reaching the colon. The slightly elevated H₂ levels seen with BAR and SCO suggest that subtle compositional differences between melon types may influence colonic fermentation.

These findings are consistent with prior reports indicating that many fruits, particularly those low in fermentable oligosaccharides, disaccharides, monosaccharides, and polyols (FODMAPs), elicit minimal H₂ production. Further investigation using larger sample sizes and complementary methods (e.g., stool metabolomics or *in vitro* fermentation models) could help clarify the fermentability and gut transit effects of different *Cucumis melo* cultivars.

A key strength of this study is its comprehensive approach, integrating population-based dietary assessments with clinically controlled physiological evaluations. The inclusion of sensory analyses, gastrointestinal tolerance, and motility measures provides a holistic understanding of how *Cucumis* vegetables influence dietary behavior and digestion. However, limitations include self-reported dietary intake and economic status may introduce reporting biases. Another limitation of the present clinical study is the relatively small sample size (*N* = 15). However, the randomized crossover design, with each participant repeating the test under all three intervention conditions, strengthened the reliability of the findings by minimizing inter-individual variability and enhancing statistical power. Nevertheless, results should be interpreted as preliminary, and further investigations involving larger and more diverse populations are warranted to confirm and extend these observations, particularly in relation to broader metabolic and health outcomes.

This study highlights the strong adherence to the MD in a well-educated, urban-dwelling population and underscores the high consumption of *Cucumis* vegetables. While these vegetables are widely accepted, differences in sensory perception, digestive tolerance, and gallbladder motility responses suggest potential implications for dietary preferences and digestive health. Understanding these factors can inform nutritional recommendations and dietary modifications to optimize individual and population health outcomes.

## 5 Conclusion

This study demonstrates the value of translational clinical approaches in evaluating how local vegetable varieties influence sensory perception, digestive physiology, and food intake regulation. By comparing the traditional Apulian *Cucumis* vegetables Barattiere and Scopatizzo with the widely consumed cucumber, we revealed clear differences in consumer experience. While cucumber consumption was linked to higher bitterness perception and greater digestive discomfort, both Barattiere and Scopatizzo were associated with more favorable sensory appeal, improved digestive tolerance, and stronger positive responses in terms of food enjoyment and intake control. These findings highlight the importance of integrating sensory evaluation, gastrointestinal response, and consumer acceptance in the nutritional assessment of regional agrobiodiversity. Evidence of this kind supports the scientific valorization of underutilized traditional crops, which not only align with consumer preferences but also contribute to gastrointestinal well-being and healthier, more sustainable dietary patterns. Future interdisciplinary research is essential to further clarify the metabolic, health, and behavioral implications of culturally relevant foods, reinforcing their role in both public health nutrition and biodiversity preservation.

## Data Availability

The raw data supporting the conclusions of this article will be made available by the authors, without undue reservation.

## Footnotes

^1^ https://sdgs.un.org/goals

^2^ ncss.com/software/ncss

## References

[1] Caso G Vecchio R. Factors influencing independent older adults (un)healthy food choices: a systematic review and research agenda. Food Res Int. (2022) 158:111476. doi: 10.1016/j.foodres.2022.111476, PMID: 35840197

[2] Willett WC Sacks F Trichopoulou A Drescher G Ferro-Luzzi A Helsing E. Mediterranean diet pyramid: a cultural model for healthy eating. Am J Clin Nutr. (1995) 61:1402s–6s. doi: 10.1093/ajcn/61.6.1402S, PMID: 7754995

[3] Sofi F Martini D Angelino D Cairella G Campanozzi A Danesi F. Mediterranean diet: why a new pyramid? An updated representation of the traditional Mediterranean diet by the Italian Society of Human Nutrition (SINU). Nutr Metab Cardiovasc Dis. (2025):103919. doi: 10.1016/j.numecd.2025.103919 40087038

[4] Marrero A Mattei J. Reclaiming traditional, plant-based, climate-resilient food systems in small islands. Lancet Planetary Health. (2022) 6:e171–9. doi: 10.1016/S2542-5196(21)00322-3, PMID: 35150626 PMC9031398

[5] de Almada CN de Nunes Almada C Martinez RC Sant'Ana Ade S. Characterization of the intestinal microbiota and its interaction with probiotics and health impacts. Appl Microbiol Biotechnol. (2015) 99:4175–99. doi: 10.1007/s00253-015-6582-5 25895093

[6] Almpounioti K Papagianni OI Koutelidakis AE. Traditional foods, the Mediterranean diet, and health promotion In: Preedy VR Patel VB, editors. Handbook of public health nutrition: International, national, and regional perspectives. Cham: Springer Nature Switzerland (2025). 1–23.

[7] Renna M Montesano FF Signore A Gonnella M Santamaria P. Biodiverso: a case study of integrated project to preserve the biodiversity of vegetable crops in Puglia (southern Italy). Agriculture. (2018) 8:128. doi: 10.3390/agriculture8080128

[8] Jaacks LM Bellows AL. Let food be thy medicine: linking local food and health systems to address the full spectrum of malnutrition in low-income and middle-income countries. BMJ Glob Health. (2017) 2:e000564. doi: 10.1136/bmjgh-2017-000564, PMID: 29225956 PMC5717933

[9] Renna M D'Imperio M Gonnella M Parente A Santamaria P Serio F. Barattiere: an Italian local variety of *Cucumis melo* L. with quality traits between melon and cucumber. Plants. (2020) 9:578. doi: 10.3390/plants9050578 PMC7284943 32370038

[10] Palmitessa OD Castellaneta A Somma A Didonna A Renna M Losito I. First report on the occurrence of Cucurbitacins in an Italian melon landrace (*Cucumis melo* L.). Horticulturae. (2023) 9. doi: 10.3390/horticulturae9111206

[11] Silva MA Albuquerque TG Alves RC Oliveira MBPP Costa HS. Melon (*Cucumis melo* L.) by-products: potential food ingredients for novel functional foods? Trends Food Sci Technol. (2020) 98:181–9. doi: 10.1016/j.tifs.2018.07.005

[12] Monteiro MP Batterham RL. The importance of the gastrointestinal tract in controlling food intake and regulating energy balance. Gastroenterology. (2017) 152:1707–17.e2. doi: 10.1053/j.gastro.2017.01.053, PMID: 28193513

[13] Furness JB Rivera LR Cho HJ Bravo DM Callaghan B. The gut as a sensory organ. Nat Rev Gastroenterol Hepatol. (2013) 10:729–40. doi: 10.1038/nrgastro.2013.180, PMID: 24061204

[14] Diella G Di Ciaula A Lorusso MP Summo C Caggiano G Caponio F. Distinct effects of two almond cultivars on agreeability and gastrointestinal motility in healthy subjects: more than mere nutraceuticals. JGLD. (2018) 27:31–9. doi: 10.15403/jgld.2014.1121.271.dll, PMID: 29557413

[15] Rizzello CG Portincasa P Montemurro M Di Palo DM Lorusso MP De Angelis M. Sourdough fermented breads are more digestible than those started with baker's yeast alone: an in vivo challenge dissecting distinct gastrointestinal responses. Nutrients. (2019) 11. doi: 10.3390/nu11122954 PMC6950244 31817104

[16] Vacca M Khalil M Rampino A Celano G Lanza E Caponio GR. Agreeability and gastrointestinal motility responses to fully characterized experimental pasta enriched in wheat by-products. J Funct Foods. (2024) 123:6598. doi: 10.1016/j.jff.2024.106598

[17] Khalil M Bonfrate L Di Ciaula A Portincasa P Abdallah H Capurso M. Self-reported symptoms after COVID-19 vaccination. Distinct sex, age, and geographical outcomes in Lebanese and Italian cohorts. Intern Emerg Med. (2023) 18:1463–75. doi: 10.1007/s11739-023-03321-9 37322183 PMC10412474

[18] Khalil M Abdallah H Calasso M Khalil N Daher A Missaoui J. Herbal medicine in three different Mediterranean living areas during the COVID-19 pandemic: the role of polyphenolic-rich thyme-like plants. Plants. (2024) 13:3340. doi: 10.3390/plants13233340, PMID: 39683135 PMC11644039

[19] Abdallah H Khalil M Farella I JohnBritto JS Lanza E Santoro S. Ramadan intermittent fasting reduces visceral fat and improves gastrointestinal motility. Eur J Clin Investig. (2023) 53:e14029. doi: 10.1111/eci.14029, PMID: 37203871

[20] Latimer Jr GW. (ed). Official methods of analysis of AOAC INTERNATIONAL. New York, NY: Oxford University Press (2023) (*22nd* Edn).

[21] Vitellio P Celano G Bonfrate L Gobbetti M Portincasa P De Angelis M. Effects of Bifidobacterium longum and *Lactobacillus rhamnosus* on gut microbiota in patients with lactose intolerance and persisting functional gastrointestinal symptoms: a randomised, double-blind, cross-over study. Nutrients. (2019) 11:886. doi: 10.3390/nu11040886, PMID: 31010241 PMC6520754

[22] Di Ciaula A Khalil M Portincasa P. Ultrasonographic assessment of gastric and gallbladder dynamics in human health and disease. Intern Emerg Med. (2025). 20, 965–983. doi: 10.1007/s11739-025-03905-7 40016490

[23] Di Ciaula A Wang DQH Portincasa P. Gallbladder and gastric motility in obese newborns, pre-adolescents and adults: age, obese and cholecysto-gastric motility. J Gastroenterol Hepatol. (2012) 27:1298–305. doi: 10.1111/j.1440-1746.2012.07149.x 22497555

[24] Portincasa P Moschetta A Berardino M Di Ciaula A Vacca M Baldassarre G. Impaired gallbladder motility and delayed orocecal transit contribute to pigment gallstone and biliary sludge formation in β -thalassemia major adults. WJG. (2004) 10:2383–90. doi: 10.3748/wjg.v10.i16.2383, PMID: 15285024 PMC4576293

[25] Di Ciaula A Covelli M Berardino M Wang DQ Lapadula G Palasciano G. Gastrointestinal symptoms and motility disorders in patients with systemic scleroderma. BMC Gastroenterol. (2008) 8:7. doi: 10.1186/1471-230X-8-7, PMID: 18304354 PMC2276219

[26] Jorge N da Silva AC Veronezi CM. Chapter 13 – antioxidant and pharmacological activity of *Cucumis melo* var. cantaloupe In: Mariod AA, editor. Multiple biological activities of unconventional seed oils. San Diego: Academic Press (2022). 147–70.

[27] Sharma V Sharma L Sandhu KS. Cucumber (*Cucumis sativus* L.) In: Nayik GA Gull A, editors. Antioxidants in vegetables and nuts – properties and health benefits. Singapore: Springer Singapore (2020). 333–40.

[28] Khan A Mishra A Hasan SM Usmani A Ubaid M Khan N. Biological and medicinal application of *Cucumis sativus* Linn. – review of current status with future possibilities. J Complement Integr Med. (2022) 19:843–54. doi: 10.1515/jcim-2020-0240, PMID: 34047145

[29] Somma A Palmitessa OD Leoni B Signore A Renna M Santamaria P. Extraseasonal production in a soilless system and characterisation of landraces of Carosello and Barattiere (*Cucumis melo* L.). Sustainability. (2021) 13:11425. doi: 10.3390/su132011425

[30] Maugeri A Barchitta M Fiore V Rosta G Favara G La Mastra C. Determinants of adherence to the Mediterranean diet: findings from a cross-sectional study in women from southern Italy. Int J Environ Res Public Health. (2019) 16:2963. doi: 10.3390/ijerph16162963, PMID: 31426512 PMC6720012

[31] Ruggiero E Di Castelnuovo A Costanzo S Persichillo M Bracone F Cerletti C. Socioeconomic and psychosocial determinants of adherence to the Mediterranean diet in a general adult Italian population. Eur J Pub Health. (2019) 29:328–35. doi: 10.1093/eurpub/cky127, PMID: 30020486

[32] Veronese N Notarnicola M Cisternino AM Inguaggiato R Guerra V Reddavide R. Trends in adherence to the Mediterranean diet in South Italy: a cross sectional study. Nutr Metabol Cardiovas Dise. (2020) 30:410–7. doi: 10.1016/j.numecd.2019.11.003, PMID: 31822430

[33] LaFlamme B. Regulation of bitterness in cucumber. Nat Genet. (2015) 47:7. doi: 10.1038/ng.3182

[34] Dai S Wang C Zhao X Ma C Fu K Liu Y. Cucurbitacin B: a review of its pharmacology, toxicity, and pharmacokinetics. Pharmacol Res. (2023) 187:106587. doi: 10.1016/j.phrs.2022.106587, PMID: 36460279

[35] Kaushik U Aeri V Mir SR. Cucurbitacins – an insight into medicinal leads from nature. Pharmacogn Rev. (2015) 9:12–8. doi: 10.4103/0973-7847.156314, PMID: 26009687 PMC4441156

[36] Chung SO Kim YJ Park SU. An updated review of cucurbitacins and their biological and pharmacological activities. EXCLI J. (2015) 14:562–6. doi: 10.17179/excli2015-283, PMID: 26648815 PMC4669946

[37] Sharma SK Puri R Jain A Sharma MP Sharma A Bohra S. Assessment of effects on health due to consumption of bitter bottle gourd (*Lagenaria siceraria*) juice Indian J Med Res. (2012) 135:49–55. doi: 10.4103/0971-5916.93424, PMID: 22382183 PMC3307184

[38] Jung C Steuber B Schwörer H. Food poisoning by cucurbitacines. Dtsch Med Wochenschr. (2020) 145:988–90. doi: 10.1055/a-1163-9741 32668470

[39] Chen JC Chiu MH Nie RL Cordell GA Qiu SX. Cucurbitacins and cucurbitane glycosides: structures and biological activities. Nat Prod Rep. (2005) 22:386–99. doi: 10.1039/b418841c, PMID: 16010347

[40] Horie H Ito H Ippoushi K Azuma K Sakata Y Igarashi I. Cucurbitacin C–bitter principle in cucumber plants. Japan Agric Res Quart. (2007) 41:65–8. doi: 10.6090/jarq.41.65

[41] Shang Y Ma Y Zhou Y Zhang H Duan L Chen H. Biosynthesis, regulation, and domestication of bitterness in cucumber. Science. (2014) 346:1084–8. doi: 10.1126/science.1259215, PMID: 25430763

[42] Liu CM Kanoski SE. Homeostatic and non-homeostatic controls of feeding behavior: distinct vs. common neural systems. Physiol Behav. (2018) 193:223–31. doi: 10.1016/j.physbeh.2018.02.011, PMID: 29421588 PMC6077115

[43] Morales I Berridge KC. ‘Liking’ and ‘wanting’ in eating and food reward: brain mechanisms and clinical implications. Physiol Behav. (2020) 227:113152. doi: 10.1016/j.physbeh.2020.113152, PMID: 32846152 PMC7655589

[44] de Wouters d’O A Huwart SJP Cani PD Everard A. Gut microbes and food reward: from the gut to the brain. Front Neurosci. (2022) 16. doi: 10.3389/fnins.2022.947240 PMC9358980 35958993

[45] Di Ciaula A Khalil M Baffy G Portincasa P. Advances in the pathophysiology, diagnosis and management of chronic diarrhoea from bile acid malabsorption: a systematic review. Eur J Intern Med. (2024) 128:10–9. doi: 10.1016/j.ejim.2024.07.008, PMID: 39069430

[46] Di Ciaula A Bonfrate L Khalil M Garruti G Portincasa P. Contribution of the microbiome for better phenotyping of people living with obesity. Rev Endo Metabol Disord. (2023) 24:839–70. doi: 10.1007/s11154-023-09798-1, PMID: 37119391 PMC10148591

[47] Portincasa P Bonfrate L Vacca M De Angelis M Farella I Lanza E. Gut microbiota and short chain fatty acids: implications in glucose homeostasis. IJMS. (2022) 23:1105. doi: 10.3390/ijms23031105, PMID: 35163038 PMC8835596

[48] Behrens M Meyerhof W. Gustatory and extragustatory functions of mammalian taste receptors. Physiol Behav. (2011) 105:4–13. doi: 10.1016/j.physbeh.2011.02.010, PMID: 21324331

[49] Kaji I Karaki S Fukami Y Terasaki M Kuwahara A. Secretory effects of a luminal bitter tastant and expressions of bitter taste receptors, T2Rs, in the human and rat large intestine. Am J Physiol Gastrointest Liver Physiol. (2009) 296:G971–81. doi: 10.1152/ajpgi.90514.2008, PMID: 19179623

[50] Rezaie P Bitarafan V Horowitz M Feinle-Bisset C. Effects of bitter substances on GI function, energy intake and glycaemia-do preclinical findings translate to outcomes in humans? Nutrients. (2021) 13:1317. doi: 10.3390/nu13041317, PMID: 33923589 PMC8072924

[51] Ho CH Ho MG Ho SP Ho HH. Bitter bottle gourd (*Lagenaria siceraria*) toxicity. J Emerg Med. (2014) 46:772–5. doi: 10.1016/j.jemermed.2013.08.106, PMID: 24360122

[52] Sofi F Macchi C Abbate R Gensini GF Casini A. Mediterranean diet and health status: an updated meta-analysis and a proposal for a literature-based adherence score. Public Health Nutr. (2014) 17:2769–82. doi: 10.1017/S1368980013003169, PMID: 24476641 PMC10282340

[53] Abdallah H Khalil M Awada E Lanza E Di Ciaula A Portincasa P. Metabolic dysfunction-associated steatotic liver disease (MASLD). Assessing metabolic dysfunction, cardiovascular risk factors, and lifestyle habits. Eur J Intern Med. (2025) 138:101–11. doi: 10.1016/j.ejim.2025.05.018, PMID: 40436716

