# Supplementary material for: Two traditional Apulian varieties of Cucumis melo L. exhibit superior sensory and digestive profiles over common cucumber: a population-based and clinical study
Source: Front Nutr. 2025 Oct 21;12:1662952. doi: 10.3389/fnut.2025.1662952 (PMC12583024; doi:10.3389/fnut.2025.1662952)
Supplement: Supplementary file 1 [file Table_1.docx]

Supplementary Material

# Supplementary Data

| Valutazione del gradimento e della digeribilità dei frutti di cetriolo, Barattiere e Scopatizzo  Questionario per i consumatori |
| --- |

Presentazione del questionario

Gent.ma/o,

Ti chiediamo di rispondere al seguente questionario che richiede circa dieci minuti del Tuo tempo.

Le risposte ottenute verranno utilizzate per completare uno studio clinico condotto presso il Policlinico di Bari e realizzato nell’ambito del progetto “ON Foods - Research and innovation network on food and nutrition Sustainability, Safety and Security—Working ON Foods”, finanziato nell’ambito del Piano Nazionale di Riprese e Resilienza (PNRR – Missione 4, Componente 2, Investimento 1.3 – Bando di gara n. 341 del 15 marzo 2022 del Ministero dell'Università e della Ricerca finanziato dall'Unione Europea – NextGenerationEU).

Il questionario ha come obiettivo la raccolta di informazioni su:

- abitudini alimentari;
- percezioni inerenti a tre tipologie di ortaggio (cetriolo, Barattiere e Scopatizzo);
- attività fisica;
- anamnesi medica.

Le risposte fornite ci aiuteranno ad effettuare una valutazione integrata in merito a gradimento e digeribilità di questi tre ortaggi, considerando sia i dati clinico-analitici raccolti durante uno studio già condotto su soggetti volontari, sia le informazioni provenienti dalla presente indagine.

I dati personali forniti saranno trattati nel rispetto del Regolamento Generale sulla Protezione dei Dati (GDPR) e di altre leggi nazionali in materia di protezione dei dati. I dati saranno utilizzati esclusivamente per scopi statistici e di ricerca, garantendo la massima riservatezza e anonimato dei partecipanti.

Grazie per la partecipazione.

| SEZIONE 1: ANAGRAFICA | |
| --- | --- |
| Sesso  ☐ Uomo  ☐ Donna  ☐ Altro | Età  ☐ < 18  ☐ 18-35  ☐ 35-64  ☐ > 64 |
| Titolo di studio  ☐ Licenza elementare  ☐ Licenza media  ☐ Diploma di scuola superiore  ☐ Laurea Triennale  ☐ Laurea Magistrale e/o titolo equipollente  ☐ Dottorato/specializzazione post-laurea | Provincia di residenza  ☐ Bari  ☐ Barletta-Andria-Trani  ☐ Brindisi  ☐ Foggia  ☐ Lecce  ☐ Taranto |
| Area di residenza  ☐ Urbana  ☐ Rurale | Condizione economica  ☐ Precaria  ☐ Instabile  ☐ Stabile  ☐ Soddisfacente  ☐ Ottimale |
| Professione  ☐ Studente  ☐ Imprenditore  ☐ Dipendente (pubblico o privato)  ☐ Libero professionista  ☐ Disoccupato  ☐ Pensionato  ☐ Altro (specificare)__________________________ | |

| SEZIONE 2: CONSUMO PRODOTTI ORTIVI |
| --- |

DOMANDA N.1 – Consumi abitualmente frutta e verdura?

☐ Sì

☐ No

DOMANDA N.2 – Quanto spesso consumi ortaggi nella tua dieta quotidiana? (1 porzione standard di ortaggi = 200 grammi; 1 porzione standard di frutta = 150 grammi; 1 porzione standard di insalate da foglia = 80 grammi; indicazioni delle linee guida per una sana alimentazione <https://www.salute.gov.it/imgs/C_17_pubblicazioni_2915_allegato.pdf>) [massimo una risposta]

☐ Raramente (≤ 1 porzione a settimana)

☐ Poco (2-4 porzioni a settimana)

☐ Abbastanza (5-8 porzioni a settimana)

☐ Frequentemente (9-12 porzioni a settimana)

☐ Tutti i giorni

DOMANDA N.3 – Conosci e segui la stagionalità dei prodotti ortivi che acquisti e consumi?

☐ Assolutamente sì

☐ Più sì che no

☐ Non saprei

☐ Più no che sì

☐ Assolutamente no

| SEZIONE 3: CONSUMO E DIGERIBILITÀ CETRIOLO (*Cucumis sativus* L.)  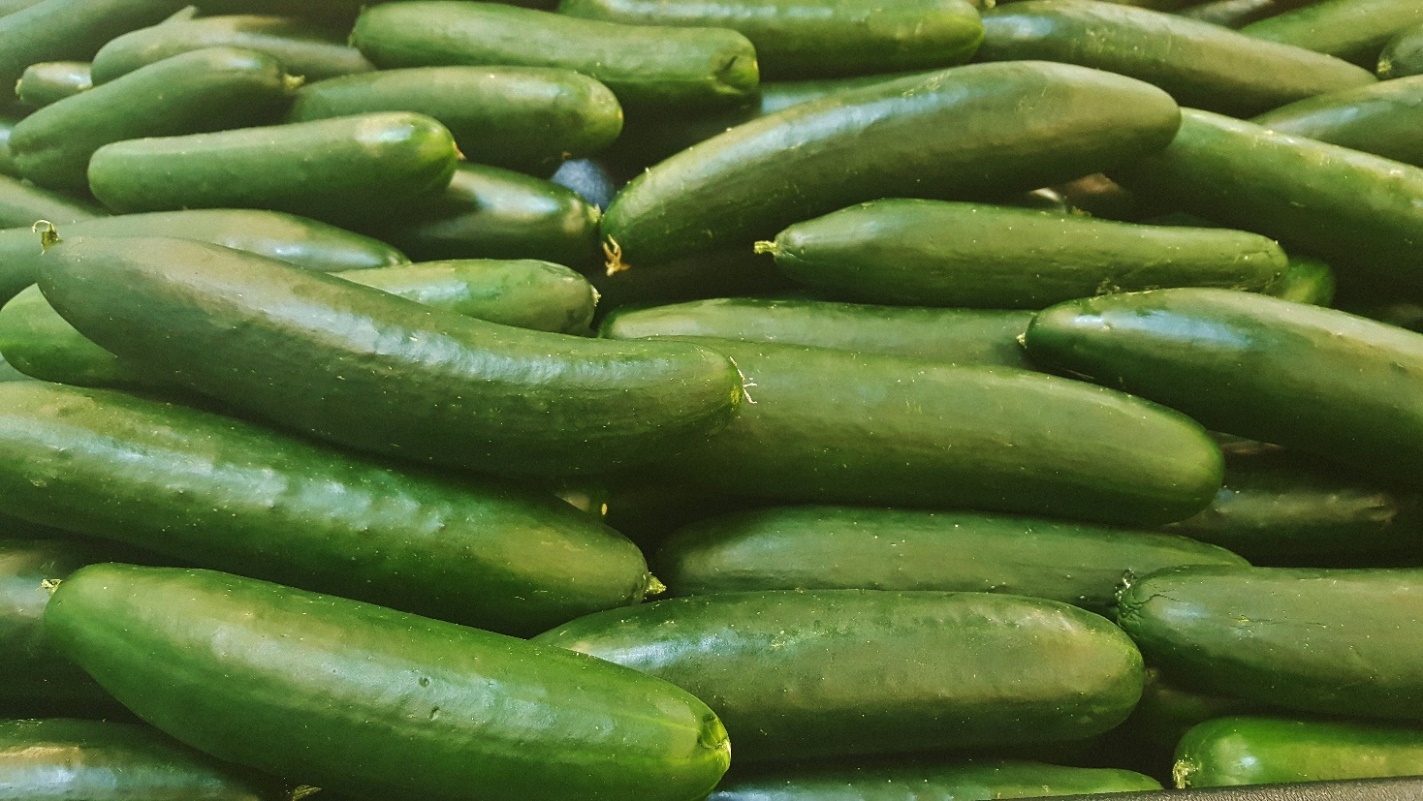  Il cetriolo ha forma allungata, simile a quella dello zucchino, buccia spessa, leggermente bitorzoluta e di colore verde, polpa bianca succosa. I semi sono distribuiti nella parte centrale. |
| --- |

DOMANDA N.4 – Conosci e acquisti abitualmente frutti di cetriolo (*Cucumis sativus* L.)?

☐ Sì, conosco e acquisto [vada a domanda N. 4]

☐ Sì, conosco ma non acquisto [vada a domanda N. 4]

☐ No, non conosco e non acquisto [vada a sezione N. 4]

DOMANDA N.4 – Con riferimento ai frutti di cetriolo, indica come valuti i seguenti parametri al consumo. [attribuire un valore da 1 (molto basso) a 5 (molto alto)]

|  | 1 2 3 4 5 |
| --- | --- |
| Dolcezza | ☐ ☐ ☐ ☐ ☐ |
| Freschezza | ☐ ☐ ☐ ☐ ☐ |
|  |  |
| Croccantezza | ☐ ☐ ☐ ☐ ☐ |
|  |  |
| Amaro | ☐ ☐ ☐ ☐ ☐ |
|  |  |
| Aroma | ☐ ☐ ☐ ☐ ☐ |
|  |  |
| Sapidità | ☐ ☐ ☐ ☐ ☐ |
|  |  |
| Succosità | ☐ ☐ ☐ ☐ ☐ |

DOMANDA N.5 – Dopo aver consumato frutti di cetriolo, hai mai avvertito sintomatologie legate a una cattiva digestione?

☐ Assolutamente sì

☐ Più sì che no

☐ Non saprei /

☐ Più no che sì

☐ Assolutamente no

DOMANDA N.6 – In caso affermativo, sapresti descrivere tali sintomi? [esempio: senso di pesantezza, gonfiore addominale, formazione di aria nello stomaco, ecc.]

________________________________________________________________________________________________________________________________________________________________________________________________________________________________________________________________________________________________________________________

| SEZIONE 4: CONSUMO E DIGERIBILITÀ BARATTIERE (*Cucumis melo* L.)  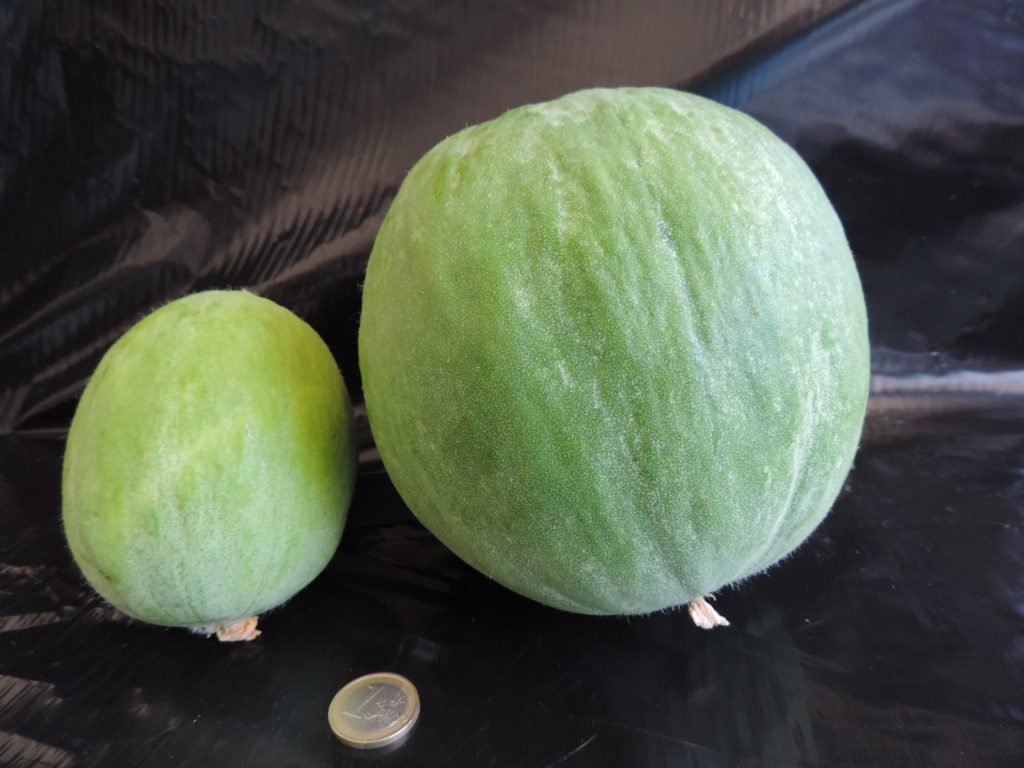  Il ‘Barattiere’ ha una forma tendenzialmente sferica, un aspetto rugoso, assenza di peluria ed un colore uniforme, variabile dal classico verde-lime al verde grigiastro. Il frutto viene commercializzato quando raggiunge un peso di 200-400 grammi. Coltivato prevalentemente nelle province di Bari e Brindisi, localmente può essere identificato con denominazioni e termini dialettali come: *barattino, cocomero, peponcine, melongedde, paddotti, spuredde* o *spureddhre* (nell'areale leccese), *scattoni, cianciuffo, pagnottella, cocomerazzo*. |
| --- |

DOMANDA N.7 – Conosci e acquisti abitualmente frutti di barattiere?

☐ Sì, conosco e acquisto [vada a domanda N .8]

☐ Sì, conosco ma non acquisto [vada a domanda N. 8]

☐ No, non conosco e non acquisto [vada a sezione N. 5]

DOMANDA N.8 – Con riferimento ai frutti di barattiere, indica come valuti i seguenti parametri al consumo. [attribuire un valore da 1 (molto basso) a 5 (molto alto)]

|  | 1 2 3 4 5 |
| --- | --- |
| Dolcezza | ☐ ☐ ☐ ☐ ☐ |
| Freschezza | ☐ ☐ ☐ ☐ ☐ |
|  |  |
| Croccantezza | ☐ ☐ ☐ ☐ ☐ |
|  |  |
| Amaro | ☐ ☐ ☐ ☐ ☐ |
|  |  |
| Aroma | ☐ ☐ ☐ ☐ ☐ |
|  |  |
| Sapidità | ☐ ☐ ☐ ☐ ☐ |
|  |  |
| Succosità | ☐ ☐ ☐ ☐ ☐ |

DOMANDA N.9 – Dopo aver consumato frutti di barattiere, hai mai avvertito sintomatologie legate a una cattiva digestione?

☐ Assolutamente sì /

☐ Più sì che no /

☐ Non saprei /

☐ Più no che sì /

☐ Assolutamente no

DOMANDA N.10 – In caso affermativo, sapresti descrivere tali sintomi? [es. senso di pesantezza, gonfiore addominale, formazione di aria nello stomaco, ecc.]

________________________________________________________________________________________________________________________________________________________________________________________________________________________________________________________________________________________________________________________

| SEZIONE 5: CONSUMO E DIGERIBILITÀ ‘SCOPATIZZO’ (*Cucumis melo* L.)  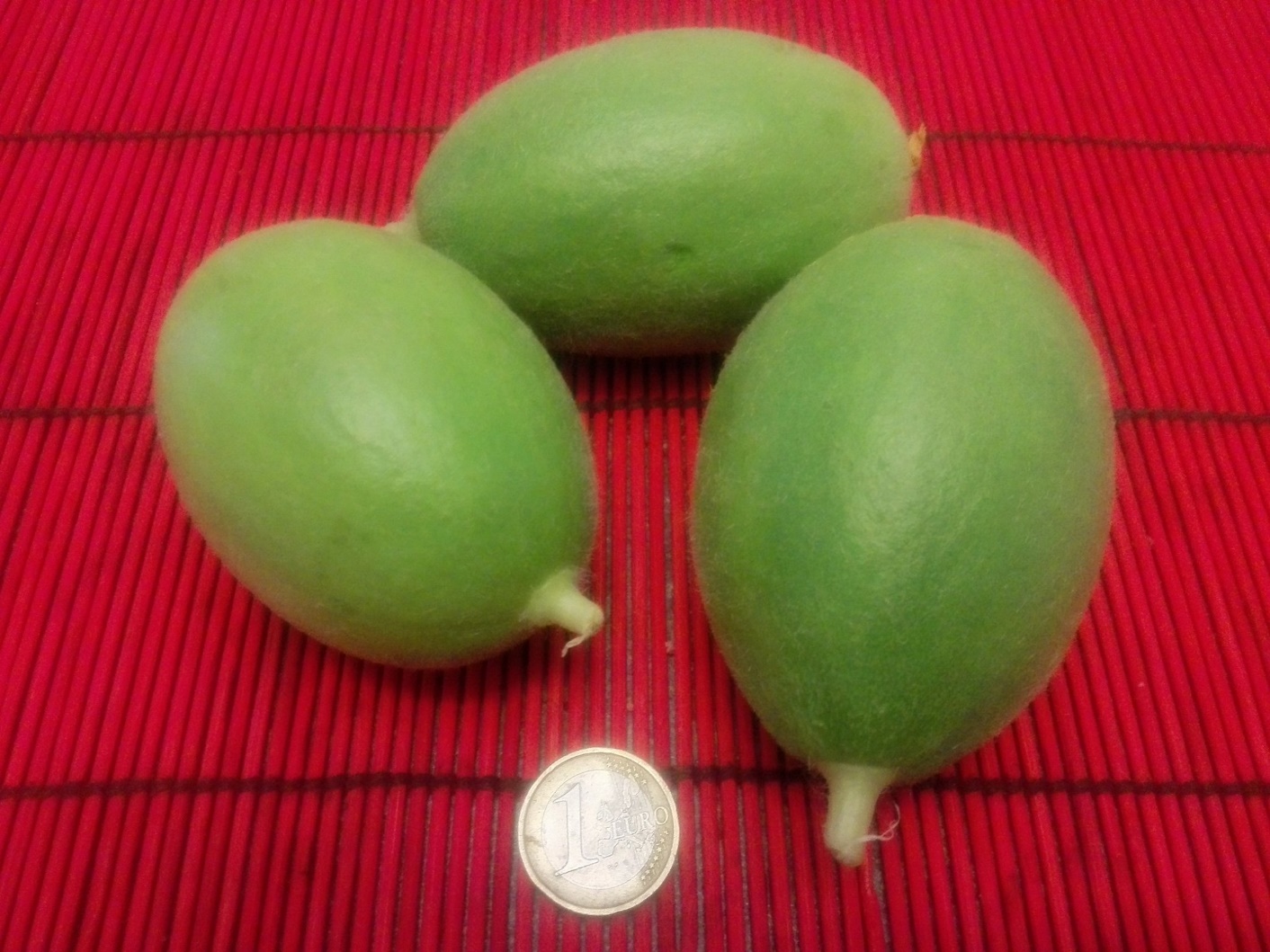  Lo ‘Scopatizzo’ è un ortaggio afferente alla stessa specie del ‘Barattiere’. Spesso, i frutti di ‘Scopatizzo’ sono confusi con quelli del ‘Barattiere’ ma, rispetto a questi ultimi, sono di forma ellittica e si caratterizzano per la dimensione piuttosto contenuta (100-120 grammi al commercio); inoltre, i frutti di ‘Scopatizzo’ sono esclusivamente di colore verde chiaro e presentano una leggera e rada tomentosità (peluria). |
| --- |

DOMANDA N.11 – Conosce e acquista abitualmente frutti di ‘Scopatizzo’?

☐ Sì, conosco e acquisto [vada a domanda N.12]

☐ Sì, conosco ma non acquisto [vada a domanda N.12]

☐ No, non conosco e non acquisto [vada a sezione N. 6]

DOMANDA N.12 – Con riferimento ai frutti di ‘Scopatizzo’, indica come valuti i seguenti parametri al consumo. [attribuire un valore da 1 (molto basso) a 5 (molto alto)]

|  | 1 2 3 4 5 |
| --- | --- |
| Dolcezza | ☐ ☐ ☐ ☐ ☐ |
| Freschezza | ☐ ☐ ☐ ☐ ☐ |
|  |  |
| Croccantezza | ☐ ☐ ☐ ☐ ☐ |
|  |  |
| Amaro | ☐ ☐ ☐ ☐ ☐ |
|  |  |
| Aroma | ☐ ☐ ☐ ☐ ☐ |
|  |  |
| Sapidità | ☐ ☐ ☐ ☐ ☐ |
|  |  |
| Succosità | ☐ ☐ ☐ ☐ ☐ |

DOMANDA N.13 – Dopo aver consumato frutti di ‘Scopatizzo’, hai mai avvertito sintomatologie legate a una cattiva digestione?

☐ Assolutamente sì

☐ Più sì che no

☐ Non saprei

☐ Più no che sì

☐ Assolutamente no

DOMANDA N.14 – In caso affermativo, sapresti descrivere tali sintomi? [es. senso di pesantezza, gonfiore addominale, formazione di aria nello stomaco, etc.]

________________________________________________________________________________________________________________________________________________________________________________________________________________________________________________________________________________________________________________________

| SEZIONE 6: ANAMNESI |
| --- |

DOMANDA N.15 – Bevi alcol?

☐ sì

☐ no

DOMANDA N.16 – Di che tipo?

☐ Vino

☐ Birra

☐ Superalcolici (liquori, amari, digestivi)

DOMANDA N.17 – Quantità di alcol a settimana?

☐ 1 bicchiere

☐ 2 bicchieri

☐ 3 bicchieri

☐ 4 bicchieri

☐ >4 bicchieri

DOMANDA N.18 – Fumi?

☐ si

☐ no

DOMANDA N.19 – Quante sigarette al giorno?

☐ 1-5

☐ 5-10

☐ 10-15

☐ 15-20

☐ >20

DOMANDA N.20 – Hai allergie/intolleranze alimentari?

☐ si

☐ no

In caso affermativo indicare quali.

__________________________________________________________________________________________________________________________________________________________________________________________________________________________________________

DOMANDA N.21 – Hai delle patologie/comorbidità?

☐ si

☐ no

In caso affermativo indicare quali.

__________________________________________________________________________________________________________________________________________________________________________________________________________________________________________

DOMANDA N.22 – Assumi quotidianamente farmaci?

☐ si

☐ no

In caso affermativo indicare quali.

__________________________________________________________________________________________________________________________________________________________________________________________________________________________________________

| SEZIONE 7: ADERENZA ALLA DIETA MEDITERRANEA – MED SCORE |
| --- |

DOMANDA N.23 – Porzioni di frutta al giorno

☐ 0-100 grammi/giorno

☐ 100-150 grammi/giorno

☐ >150 grammi/giorno

DOMANDA N.24 – Porzioni di verdura al giorno

☐ 0-100 grammi/giorno

☐ 100-250 grammi/giorno

☐ >250 grammi/giorno

DOMANDA N.25 – Porzioni di legumi a settimana

☐ 0-70 grammi/settimana

☐ 70-140 grammi/settimana

☐ >140 grammi/settimana

DOMANDA N.26 – Porzioni di cereali al giorno

☐ 0-130 grammi/giorno

☐ 130-200 grammi/giorno

☐ >200 grammi/giorno

DOMANDA N.37 – Porzioni di pesce a settimana

☐ 0-100 grammi/settimana

☐ 100-250 grammi/settimana

☐ >250 grammi/settimana

DOMANDA N.28 – Porzioni di carne e prodotti a base di carne al giorno

☐ 0-80 grammi/giorno

☐ 80-120 grammi/giorno

☐ >120 grammi/giorno

DOMANDA N.32 – Porzioni di prodotti lattiero-caseari al giorno

☐ 0-180 grammi/giorno

☐ 180-270 grammi/giorno

☐ >270 grammi/giorno

DOMANDA N.29 – Alcol (1 bevanda= 12 g di alcol)

☐ <1 bevanda/giorno

☐ 1-2 bevande/giorno

☐ >2 bevande/giorno

DOMANDA N.30 – Consumo di olio d’oliva

☐ occasionale

☐ frequente

☐ regolare

| SEZIONE 8: Attività fisica  Siamo interessati a conoscere i tipi di attività fisica che le persone fanno come parte della vita quotidiana. Le domande riguarderanno il tempo che tu hai trascorso in attività fisiche negli ultimi sette giorni. Cortesemente, rispondi ad ogni domanda anche se non ti consideri una persona attiva. Pensa, per favore, alle attività svolte sia al lavoro, che in casa, in giardino, per spostarsi da un luogo all’altro che nel tuo tempo libero come divertimento, esercizio fisico o sport.  Nel rispondere alle domande tieni in considerazione i seguenti criteri;  • Per INTENSA attività fisica si intende una attività che richiede uno sforzo fisico elevato e che la costringe a respirare con un ritmo molto più elevato del normale.  • Per MODERATA attività si intende una attività che richiede uno sforzo fisico moderato e che la costringe a respirare con un ritmo solo moderatamente più elevato del normale.  Nel rispondere alle domande, tieni conto solo di quelle attività che ti hanno impegnata por almeno 10 minuti ogni volta.  N.B. Rispondi una sola volta per ogni domanda, e segui le eventuali istruzioni tra parentesi. |
| --- |

DOMANDA N.31 - Negli ultimi 7 giorni, per quanti giorni hai compiuto attività fisiche INTENSE, come ad esempio sollevamento di pesi, lavori pesanti in giardino, attività aerobiche come corse o giri in bicicletta a velocità sostenuta?

☐ 1

☐ 2

☐ 3

☐ 4

☐ 5

☐ 6

☐ 7

☐ nemmeno 1 (vai alla domanda 33)

DOMANDA N.32 – Quanto tempo in totale di solito trascorri in attività fisiche INTENSE in uno di questi giorni?

Numero minuti: _____

DOMANDA N.33 – Negli ultimi 7 giorni, per quanti giorni hai compiuto attività fisiche MODERATE, come ad esempio trasporto di pesi leggeri, giri in bicicletta ad una velocità regolare, attività in palestra, lavoro in giardino, lavoro fisico prolungato in casa? Non considerare le camminate.

☐ 1

☐ 2

☐ 3

☐ 4

☐ 5

☐ 6

☐ 7

☐ nemmeno 1 (vai alla domanda 35)

DOMANDA N.34 – Quanto tempo in totale di solito trascorri in attività fisiche MODERATE in uno di questi giorni?

Numero minuti: _____

DOMANDA N.35 – Negli ultimi 7 giorni, per quanti giorni hai CAMMINATO per almeno 10 minuti ogni volta? Considera le camminate compiute al lavoro e a casa, quelle per spostarsi da un posto ad un altro, ed ogni altra camminata che ti è capitato di fare anche solo per piacere, esercizio o sport.

☐ 1

☐ 2

☐ 3

☐ 4

☐ 5

☐ 6

☐ 7

☐ nemmeno 1 (vai alla domanda 38)

DOMANDA N.36 – Quanto tempo in totale di solito trascorri in CAMMINATE in uno di questi giorni?

Numero minuti: _____

DOMANDA N.37 – A che passo hai CAMMINATO prevalentemente?

☐ passo INTENSO, che ti ha fatto respirare ad un ritmo molto più elevato del normale

☐ passo MODERATO, che ti ha fatto respirare ad un ritmo solo moderatamente più elevato del normale

☐ passo LENTO, tale che non c’è stato nessun cambiamento nel tuo ritmo di respiro

DOMANDA N.38 – Nell’ultima settimana lavorativa (dal lunedì al venerdì) quanti minuti al giorno, mediamente, hai trascorso rimanendo seduto?

Numero minuti: _____

DOMANDA N.39 – Nell’ultimo fine-settimana (sabato e domenica) quanti minuti al giorno, mediamente, hai trascorso rimanendo seduto?

Numero minuti: _____

| Assessment of Consumer Appreciation and Digestibility of Cucumber, Barattiere, and Scopatizzo Fruits  Consumer Questionnaire |
| --- |

Questionnaire Introduction

Dear Participant,

We kindly ask you to complete the following questionnaire, which will take approximately ten minutes of your time.

The responses will be used in a clinical study conducted at the Policlinico of Bari, within the project “ON Foods – Research and innovation network on food and nutrition Sustainability, Safety and Security—Working ON Foods”, funded under the Italian National Recovery and Resilience Plan (PNRR – Mission 4, Component 2, Investment 1.3 – Call No. 341 dated March 15, 2022, from the Ministry of University and Research, funded by the European Union – NextGenerationEU).

The purpose of the questionnaire is to collect information on:

- dietary habits;
- perceptions regarding three types of vegetables (cucumber, Barattiere, and Scopatizzo);
- physical activity;
- medical history.

Your answers will help us to perform an integrated evaluation of consumer appreciation and digestibility of the three vegetables, combining clinical-analytical data from a study already conducted on volunteers with data collected from this survey.

All personal data will be treated in accordance with the General Data Protection Regulation (GDPR) and other national data protection laws. The data will be used exclusively for statistical and research purposes, ensuring full confidentiality and anonymity of the participants.

Thank you for your participation.

| SECTION 1: DEMOGRAPHICS | |
| --- | --- |
| Gender ☐ Male  ☐ Female  ☐ Other | Age ☐ < 18  ☐ 18–35  ☐ 35–64  ☐ > 64 |
| Education ☐ Primary school ☐ Middle school ☐ High school diploma ☐ Bachelor's degree ☐ Master's degree or equivalent ☐ PhD/Postgraduate specialization | Province of residence  ☐ Bari  ☐ Barletta-Andria-Trani  ☐ Brindisi  ☐ Foggia  ☐ Lecce  ☐ Taranto |
| Area of residence  ☐ Urban  ☐ Rural | Economic condition  ☐ Precarious  ☐ Unstable  ☐ Stable  ☐ Satisfactory  ☐ Optimal  Profession |
| Profession ☐ Student  ☐ Entrepreneur  ☐ Employee (public or private)  ☐ Freelancer  ☐ Unemployed  ☐ Retired  ☐ Other (please specify) _______________ | |

| SECTION 2: VEGETABLE CONSUMPTION |
| --- |

Q1 – Do you usually eat fruit and vegetables?

☐ Yes

☐ No

Q2 – How often do you consume vegetables in your daily diet?
(1 standard portion = 200g vegetables, 150g fruit, 80g leafy salad; per healthy eating guidelines)
☐ Rarely (≤ 1 portion/week)

☐ Occasionally (2–4 portions/week)

☐ Moderately (5–8 portions/week)

☐ Frequently (9–12 portions/week)

☐ Daily

Q3 – Are you aware of and do you follow the seasonality of the vegetables you buy and eat?
☐ Absolutely yes

☐ More yes than no

☐ I don’t know

☐ More no than yes

☐ Absolutely no

| SECTION 3: CUCUMBER CONSUMPTION AND DIGESTIBILITY (*Cucumis sativus* L.)  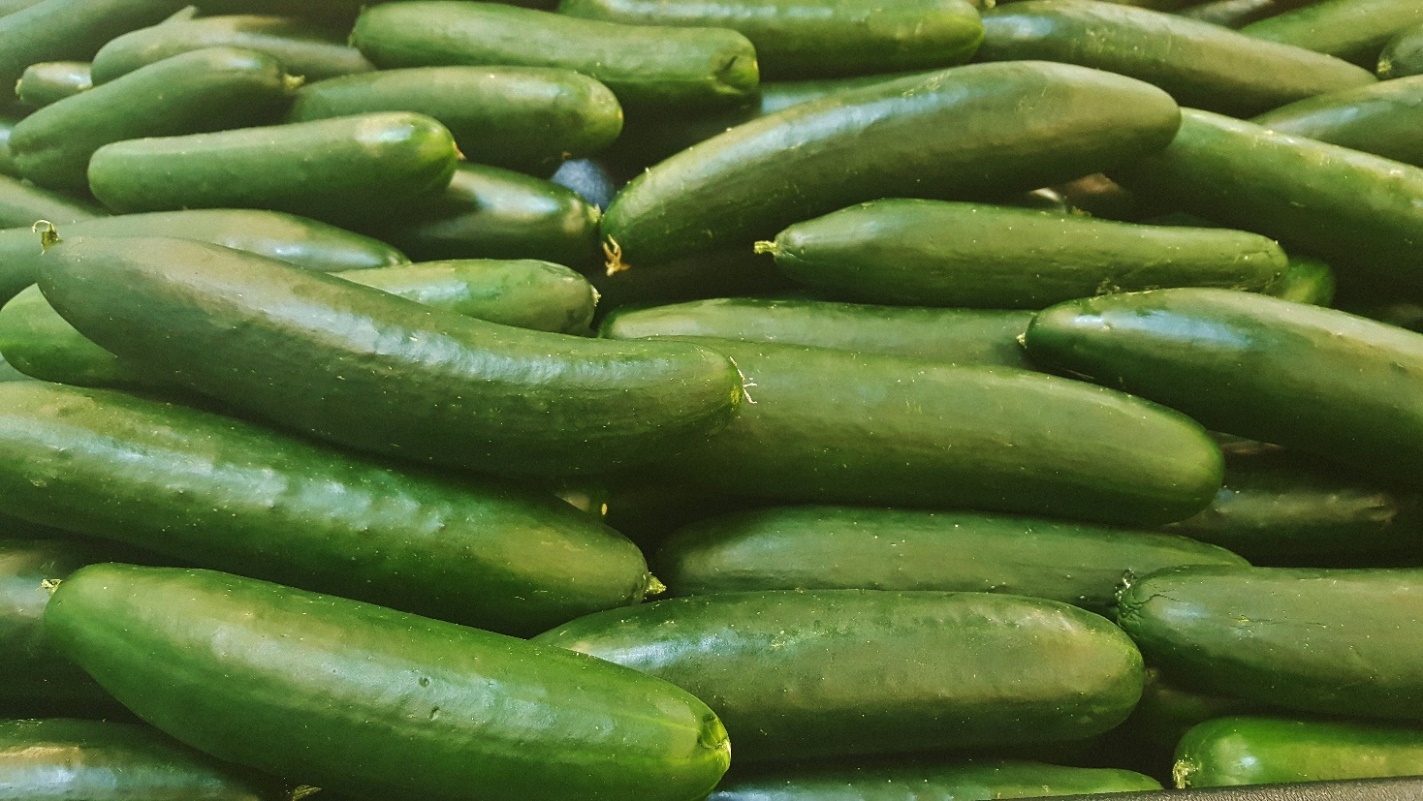  Cucumber (Cucumis sativus L.) is long-shaped, similar to zucchini, with thick slightly bumpy green skin and juicy white flesh. Seeds are central.. |
| --- |

Q4 – Are you familiar with and do you purchase cucumbers?
☐ Yes, I know and buy them

☐ Yes, I know but do not buy them

☐ No, I do not know or buy them

Q5 – Please rate the following cucumber qualities on a scale of 1 (very low) to 5 (very high):

|  | 1 2 3 4 5 |
| --- | --- |
| Sweetness | ☐ ☐ ☐ ☐ ☐ |
| Freshness | ☐ ☐ ☐ ☐ ☐ |
|  |  |
| Crunchiness | ☐ ☐ ☐ ☐ ☐ |
|  |  |
| Bitterness | ☐ ☐ ☐ ☐ ☐ |
|  |  |
| Aroma | ☐ ☐ ☐ ☐ ☐ |
|  |  |
| Saltiness | ☐ ☐ ☐ ☐ ☐ |
|  |  |
| Juiciness | ☐ ☐ ☐ ☐ ☐ |

Q6 – Have you ever experienced digestive issues after consuming cucumbers?
☐ Absolutely yes

☐ More yes than no

☐ Not sure

☐ More no than yes

☐ Absolutely no

Q7 – If yes, please describe the symptoms (e.g., heaviness, bloating, gas, etc.):________________________________________________________________________________________________________________________________________________________________________________________________________________________________________________________________________________________________________________________

| SECTION 4: BARATTIERE CONSUMPTION AND DIGESTIBILITY (*Cucumis melo* L.)  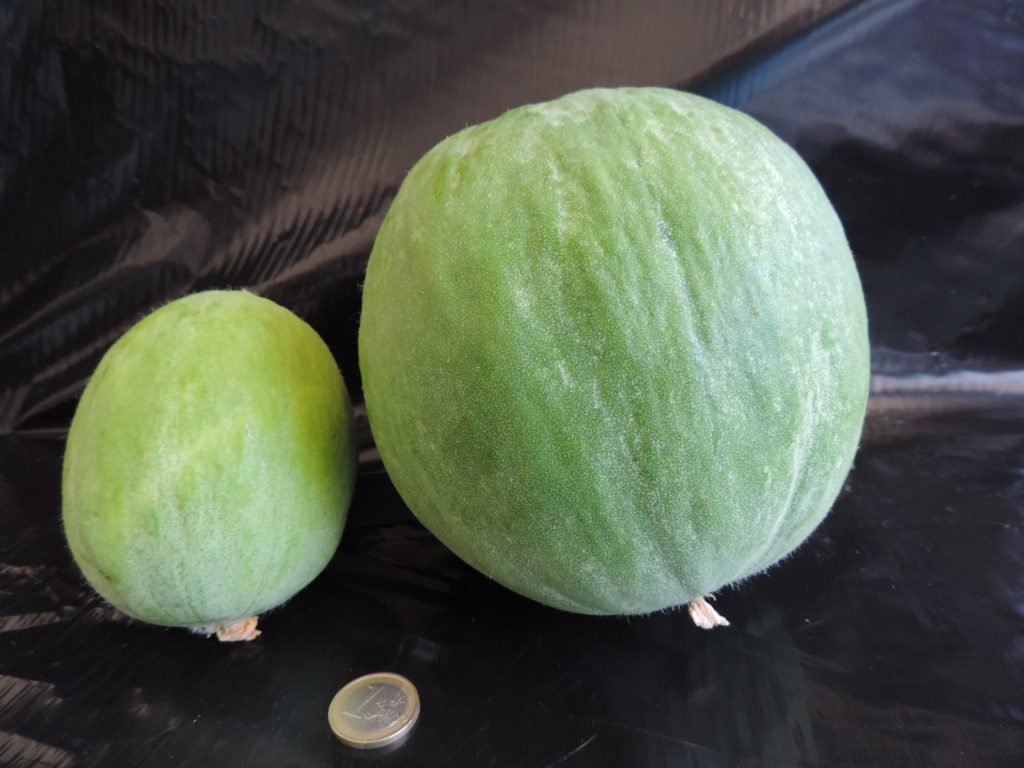  Barattiere (Cucumis melo L.) is generally spherical, wrinkled, hairless, uniformly green (lime to greyish), and typically weighs 200–400g. Mainly grown in Bari and Brindisi, it has many local names like “barattino,” “spureddhre,” or “cianciuffo. |
| --- |

Q8 – Are you familiar with and do you purchase Barattiere?
☐ Yes, I know and buy it

☐ Yes, I know but do not buy it

☐ No, I do not know or buy it

Q9- Please rate the following Barattiere qualities on a scale of 1 (very low) to 5 (very high):

|  | 1 2 3 4 5 |
| --- | --- |
| Sweetness | ☐ ☐ ☐ ☐ ☐ |
| Freshness | ☐ ☐ ☐ ☐ ☐ |
|  |  |
| Crunchiness | ☐ ☐ ☐ ☐ ☐ |
|  |  |
| Bitterness | ☐ ☐ ☐ ☐ ☐ |
|  |  |
| Aroma | ☐ ☐ ☐ ☐ ☐ |
|  |  |
| Saltiness | ☐ ☐ ☐ ☐ ☐ |
|  |  |
| Juiciness | ☐ ☐ ☐ ☐ ☐ |

Q10 – Have you ever experienced digestive issues after consuming Barattiere?
☐ Absolutely yes
☐ More yes than no
☐ Not sure
☐ More no than yes
☐ Absolutely no

Q11 – If yes, please describe the symptoms:

________________________________________________________________________________________________________________________________________________________________________________________________________________________________________________________________________________________________________________________

| SECTION 5: SCOPATIZZO CONSUMPTION AND DIGESTIBILITY (*Cucumis melo* L.)  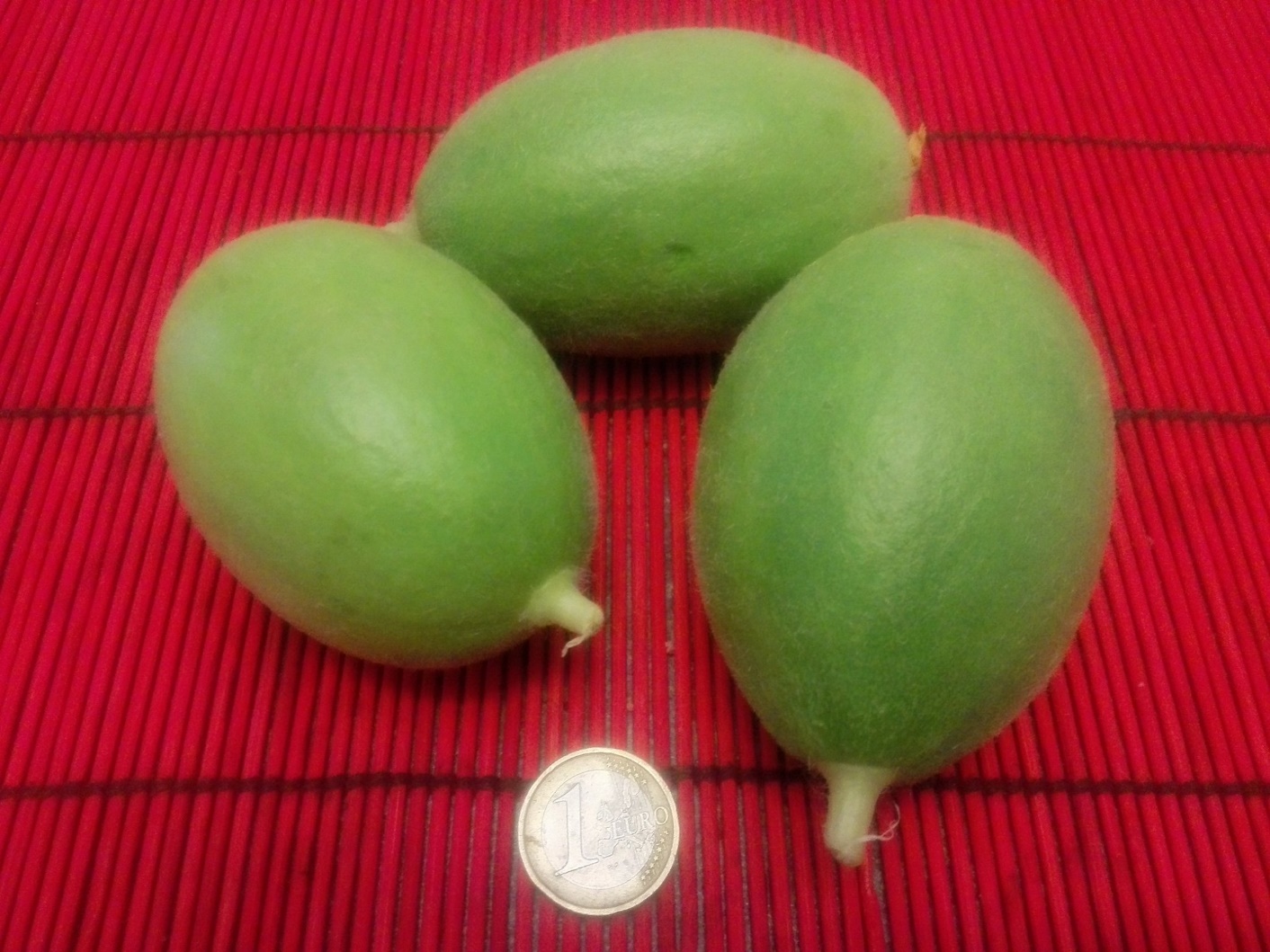  Scopatizzo (Cucumis melo L.) is of the same species as Barattiere but is smaller (100–120g), elliptical, light green, and slightly hairy. |
| --- |

Q12 – Are you familiar with and do you purchase Scopatizzo?
☐ Yes, I know and buy it

☐ Yes, I know but do not buy it

☐ No, I do not know or buy it

Q13 – Please rate the following Scopatizzo qualities on a scale of 1 (very low) to 5 (very high):

|  | 1 2 3 4 5 |
| --- | --- |
| Sweetness | ☐ ☐ ☐ ☐ ☐ |
| Freshness | ☐ ☐ ☐ ☐ ☐ |
|  |  |
| Crunchiness | ☐ ☐ ☐ ☐ ☐ |
|  |  |
| Bitterness | ☐ ☐ ☐ ☐ ☐ |
|  |  |
| Aroma | ☐ ☐ ☐ ☐ ☐ |
|  |  |
| Saltiness | ☐ ☐ ☐ ☐ ☐ |
|  |  |
| Succosità | ☐ ☐ ☐ ☐ ☐ |

Q14 – Have you ever experienced digestive issues after consuming Scopatizzo?
☐ Absolutely yes
☐ More yes than no
☐ Not sure
☐ More no than yes
☐ Absolutely no

Q15 – If yes, please describe the symptoms

________________________________________________________________________________________________________________________________________________________________________________________________________________________________________________________________________________________________________________________

| SECTION 6: MEDICAL HISTORY |
| --- |

QUESTION 15 – Do you drink alcohol?

☐ Yes

☐ No

QUESTION 16 – What type?

☐ Wine

☐ Beer

☐ Spirits (liquors, bitters, digestifs)

QUESTION 17 – Amount of alcohol per week?

☐ 1 glass

☐ 2 glasses

☐ 3 glasses

☐ 4 glasses

☐ More than 4 glasses

QUESTION 18 – Do you smoke?

☐ Yes

☐ No

QUESTION 19 – How many cigarettes per day?

☐ 1–5

☐ 5–10

☐ 10–15

☐ 15–20

☐ More than 20

QUESTION 20 – Do you have any food allergies/intolerances?
☐ Yes

☐ No

If yes, please specify:

QUESTION 21 – Do you have any diseases/comorbidities?
☐ Yes

☐ No

If yes, please specify:

.

__________________________________________________________________________________________________________________________________________________________________________________________________________________________________________

QUESTION 22 – Do you take any medications daily?

☐ Yes

☐ No

If yes, please specify:

__________________________________________________________________________________________________________________________________________________________________________________________________________________________________________

| SECTION 7: ADHERENCE TO THE MEDITERRANEAN DIET – MED SCORE |
| --- |

QUESTION 23 – Fruit servings per day

☐ 0–100 grams/day

☐ 100–150 grams/day

☐ More than 150 grams/day

QUESTION 24 – Vegetable servings per day

☐ 0–100 grams/day

☐ 100–250 grams/day

☐ More than 250 grams/day

QUESTION 25 – Legume servings per week

☐ 0–70 grams/week

☐ 70–140 grams/week

☐ More than 140 grams/week

QUESTION 26 – Cereal servings per day

☐ 0–130 grams/day

☐ 130–200 grams/day

☐ More than 200 grams/day

QUESTION 27 – Fish servings per week

☐ 0–100 grams/week

☐ 100–250 grams/week

☐ More than 250 grams/week

QUESTION 28 – Meat and meat-based product servings per day

☐ 0–80 grams/day

☐ 80–120 grams/day

☐ More than 120 grams/day

QUESTION 29 – Dairy product servings per day

☐ 0–180 grams/day

☐ 180–270 grams/day

☐ More than 270 grams/day

QUESTION 30 – Alcohol (1 drink = 12 g of alcohol)

☐ Less than 1 drink/day

☐ 1–2 drinks/day

☐ More than 2 drinks/day

QUESTION 31 – Olive oil consumption

☐ Occasional

☐ Frequent

☐ Regular

| SECTION 8: PHYSICAL ACTIVITY  We are interested in learning about the types of physical activity people do as part of their daily lives. The questions refer to the time you spent doing physical activities in the last seven days. Please answer every question, even if you do not consider yourself an active person. Think about activities done at work, at home, in the garden, for transportation, and in your leisure time, including fun, exercise, or sports.  When answering the questions, consider the following:   - VIGOROUS physical activity refers to activities that require high physical effort and make you breathe much harder than normal. - MODERATE physical activity refers to activities that require moderate physical effort and make you breathe somewhat harder than normal.   Only include activities that lasted at least 10 minutes at a time. Note: Answer each question only once, and follow any instructions in parentheses |
| --- |

QUESTION 32 – In the last 7 days, on how many days did you do VIGOROUS physical activities, such as weightlifting, heavy gardening, aerobics, running, or fast cycling?
☐ 1
☐ 2
☐ 3
☐ 4
☐ 5
☐ 6
☐ 7
☐ None (go to question 34)

QUESTION 33 – How much time in total do you usually spend doing VIGOROUS physical activities on one of those days?
Number of minutes: _____

QUESTION 34 – In the last 7 days, on how many days did you do MODERATE physical activities, such as carrying light loads, cycling at a regular pace, gym activities, gardening, or prolonged housework? Do not include walking.
☐ 1
☐ 2
☐ 3
☐ 4
☐ 5
☐ 6
☐ 7
☐ None (go to question 36)

QUESTION 35 – How much time in total do you usually spend doing MODERATE physical activities on one of those days?
Number of minutes: _____

QUESTION 36 – In the last 7 days, on how many days did you WALK for at least 10 minutes at a time? Include walking at work, at home, for travel, and any walking done for fun, exercise, or sport.
☐ 1
☐ 2
☐ 3
☐ 4
☐ 5
☐ 6
☐ 7
☐ None (go to question 39)

QUESTION 37 – How much time in total do you usually spend WALKING on one of those days?
Number of minutes: _____

QUESTION 38 – At what pace did you mostly WALK?
☐ VIGOROUS pace, making you breathe much harder than normal
☐ MODERATE pace, making you breathe somewhat harder than normal
☐ SLOW pace, with no change in breathing rate

QUESTION 39 – During the last workweek (Monday to Friday), how many minutes per day on average did you spend sitting?
Number of minutes: _____

QUESTION 40 – During the last weekend (Saturday and Sunday), how many minutes per day on average did you spend sitting?
Number of minutes: _____
